# MAP4K3/GLK inhibits Treg differentiation by direct phosphorylating IKKβ and inducing IKKβ-mediated FoxO1 nuclear export and Foxp3 downregulation

**DOI:** 10.7150/thno.72148

**Published:** 2022-07-18

**Authors:** Jyun-Ni Chi, Jhih-Yu Yang, Chia-Hsin Hsueh, Ching-Yi Tsai, Huai-Chia Chuang, Tse-Hua Tan

**Affiliations:** 1 Immunology Research Center, National Health Research Institutes, Zhunan, Taiwan.; 2 Department of Pathology & Immunology, Baylor College of Medicine, Houston, Texas, USA.

**Keywords:** MAP4K3/GLK, FoxO1, IKKβ, Foxp3, Treg

## Abstract

**Rationale:** GLK (MAP4K3) activates PKCθ-IKKβ axis in T-cell activation and induces IL-17A-mediated autoimmune diseases. Attenuation of Treg differentiation and function by GLK could also contribute to autoimmune diseases.

**Methods:** We analyzed the roles of GLK and IKKβ in Treg differentiation and function using T-cell-specific GLK transgenic mice and IKKβ conditional knockout mice. The mechanism of GLK/IKKβ-mediated attenuation of Treg differentiation/function was studied by chromatin-immunoprecipitation, reporter assays, *in vitro* kinase assays, protein-protein interaction assays, mass spectrometry, confocal microscopy, flow cytometry, and single-cell RNA sequencing (scRNA-seq) analysis.

**Results:** We found that GLK signaling inhibited Foxp3 transcription by blocking the function of the transcription factor FoxO1. Mechanistically, GLK directly phosphorylated and activated IKKβ at Ser733 in a PKCθ-independent manner. The phospho-IKKβ Ser733 induced FoxO1 Ser319 phosphorylation and nuclear export, leading to Foxp3 downregulation. Consistently, scRNA-seq analyses showed that Foxp3 mRNA levels were inversely correlated with FoxO1 mRNA levels in GLK transgenic CD4^+^ T cells.

**Conclusions:** GLK-IKKβ-FoxO1 signaling axis inhibits Foxp3 transcription, leading to reduction of Treg differentiation and suppressive activity, as well as induction of autoimmune disease.

## Introduction

Regulatory T (Treg) cells exert their immunosuppressive functions for maintaining homeostasis, promoting self-tolerance, and suppressing inflammation 1, 2. Natural Treg (nTreg) cells differentiate in the thymus as a distinct lineage, consisting 2-4% of CD4 single-positive thymocytes 3. Peripherally generated adaptive Treg (a.k.a. induced Treg [iTreg]) cells are originated from Foxp3^-^CD4^+^ T cells 4. iTreg cells lack neuropilin-1 (Nrp-1) expression under tolerogenic environment; Nrp-1 distinguishes iTreg cells from nTreg cells in mice 5. The forkhead box transcription factor Foxp3 is crucial for the establishment of Treg-cell development, function, and stability 1, 2. Disruptive mutations in the *Foxp3* gene result in severe and early-onset autoimmunity with a scurfy phenotype in mice and with IPEX (immune dysregulation, polyendocrinopathy, enteropathy, X-linked) syndrome in humans 6, 7.

GLK (also named MAP4K3) 8 protein levels are increased in T cells of patients with systemic lupus erythematosus (SLE) 9, 10, rheumatoid arthritis (RA) 11, and adult-onset Still's disease (AOSD) 12. GLK overexpression selectively promotes IL-17A transcription in GLK transgenic (Tg) T cells and in human autoimmune T cells 13, 14. T-cell-specific GLK Tg mice display normal T-cell development and spontaneously develop Th17-mediated autoimmune diseases 13. Conversely, GLK-deficient mice exhibit resistance to experimental autoimmune encephalomyelitis (EAE) induction 9. GLK-deficient regulatory T (Treg) cells show enhanced suppressive function compared to those of wild-type Treg cells 9.

The forkhead box class O (FoxO) family in mammals is comprised of the evolutionally highly conserved forkhead transcription factors, FoxO1, FoxO3a, FoxO4, and FoxO6 15. FoxO transcription factors play crucial roles in regulating immune-cell functions, including immune homeostasis and cellular differentiation 16. FoxO1 is highly expressed in lymphoid cells, especially in T and B cells 17; moreover, FoxO3a is widely expressed in multiple tissues including lymphoid and myeloid lineage cells 18, 19. *In vitro* differentiated-iTreg cells show markedly reduction of Foxp3 protein levels in FoxO1 knockout or FoxO1/FoxO3a double conditional knockout T cells 20, 21. The transcription factors FoxO1 and FoxO3a bind directly to the Foxp3 promoter and up-regulate Foxp3 expression 20, 21. The subcellular localization and transcriptional activity of FoxO1 and FoxO3a are regulated by phosphorylation 22, 23. For example, IKKβ induces the FoxO3a Ser644 phosphorylation in cancer cells, leading to the nuclear export of FoxO3a 24, 25. Moreover, Thr24 phosphorylation of FoxO1 is decreased in Treg cells upon TCR stimulation compared to that of conventional T cells, resulting in the nuclear retention of FoxO1 26. However, it is unclear whether GLK signaling induces FoxO1/FoxO3a phosphorylation, leading to dysfunction of Treg cells.

To investigate the mechanism of GLK signaling-induced attenuation of Treg function, we analyzed T-cell specific GLK Tg mice and T cells, as well as IKKβ conditional knockout mice. Here, we report that GLK downregulates Foxp3 transcription by inducing IKKβ-mediated FoxO1 phosphorylation.

## Results

### GLK signaling inhibits Treg differentiation and suppressive function through Foxp3 downregulation

**Figure 1:**
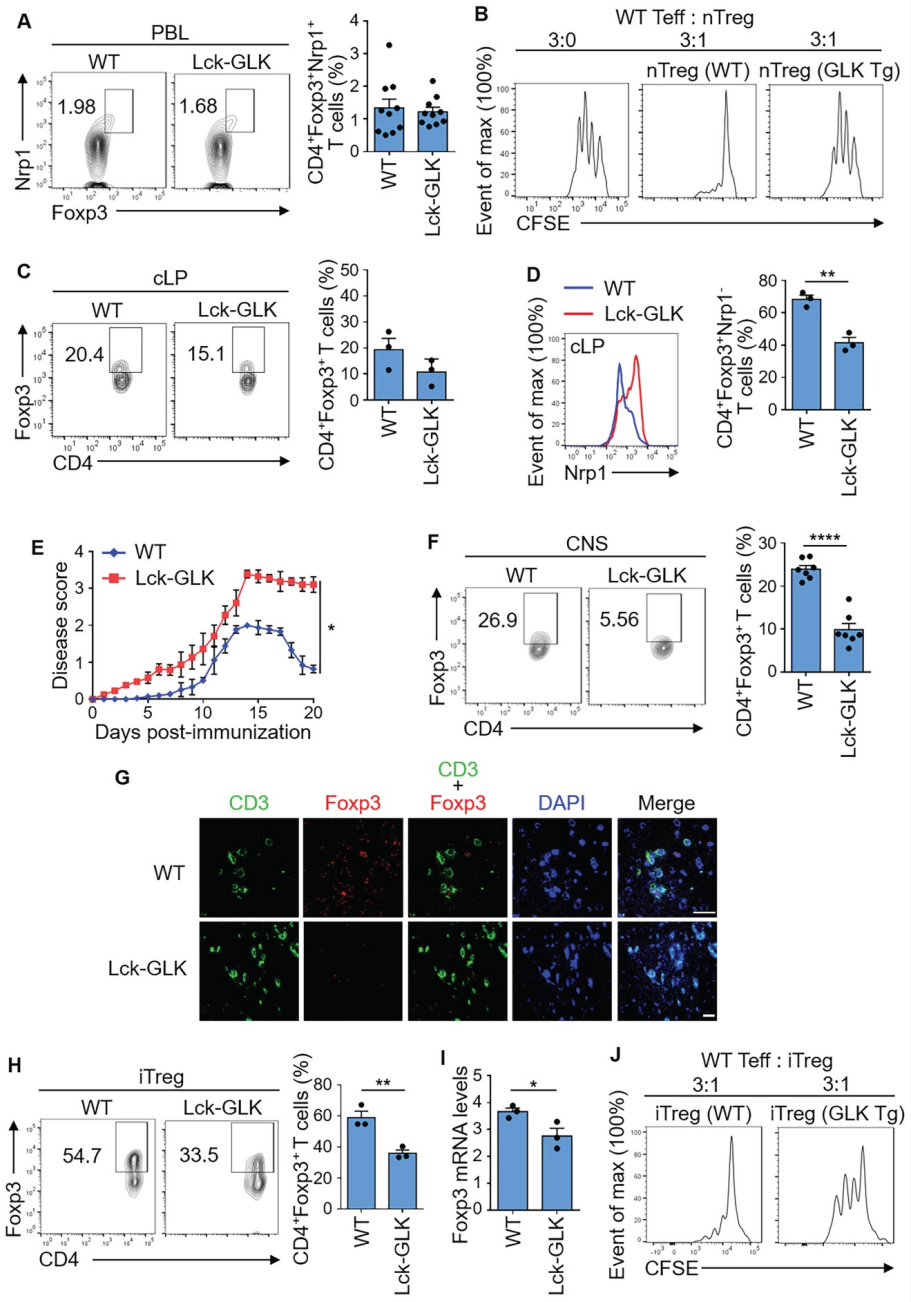
** GLK transgene inhibits Treg differentiation and suppressive function. (A)** Flow cytometry plots represent the frequency of Foxp3^+^Nrp-1^+^ cells in gated CD4^+^ T cells from peripheral blood of wild-type (WT) and Lck-GLK transgenic (Tg) mice (n=10 per group), with a quantitative graph of nTregs (natural Treg; CD4^+^Foxp3^+^Nrp-1^+^ T cells) among CD4^+^ T cells from peripheral blood. Mean ± SEM are shown.** (B)** Suppression of CFSE-labeled effector T cells (Teff) by nTreg cells of WT or Lck-GLK Tg mice. nTreg cells were cocultured with Teff cells at a ratio of 1:3 in the anti-CD3 antibody-coated plates. **(C)** Flow cytometry plots represent the frequency of CD4^+^Foxp3^+^ cells in gated CD4^+^ T cells from the colon lamina propria (cLP) of WT and Lck-GLK Tg mice (n=3 per group), with a quantitative graph of CD4^+^Foxp3^+^ T cells among CD4^+^ T cells from the cLP. Mean ± SEM are shown.** (D)** Representative histogram of Nrp-1 expression gated on CD4^+^Foxp3^+^ T cells from the cLP of WT and Lck-GLK Tg mice (n=3 per group), with a quantitative graph of iTregs (induced Treg; CD4^+^Foxp3^+^Nrp-1^-^ T cells) among CD4^+^Foxp3^+^ T cells from the cLP. Mean ± SEM are shown.** (E)** EAE clinical scores using the scale of 1 to 5 in groups of Lck-GLK Tg mice and their WT littermates (n=6 per group) after EAE induction. **(F)** Flow cytometry plots represent the frequency of CD4^+^Foxp3^+^ cells in gated CD4^+^ T cells from the central nervous system (CNS) of WT and Lck-GLK Tg mice at day 20 after EAE induction, with a quantitative graph of CD4^+^Foxp3^+^ T cells among CD4^+^ T cells from the CNS. Mean ± SEM are shown.** (G)** Representative images of the confocal microscopy analysis of CD3^+^ (green), Foxp3^+^ (Red), and DAPI (blue) in the section of the brain from WT and Lck-GLK Tg mice at day 20 after EAE induction. Original magnification, x630; scale bars, 25 μm. **(H)** Flow cytometry analysis of CD4^+^Foxp3^+^ T cells among *in vitro*-differentiated iTreg cells from WT and Lck-GLK Tg mice with a quantitative graph of CD4^+^Foxp3^+^ T cells among CD4^+^ T cells. **(I)** Real-time PCR of Foxp3 mRNA levels in *in vitro*-differentiated iTreg (CD4^+^Foxp3^+^) cells from WT and Lck-GLK Tg mice. Foxp3 mRNA levels were normalized to Srp72 mRNA levels. Mean ± SEM are shown. **(J)** Suppression of CFSE-labeled Teff by WT or GLK Tg iTreg cells. iTreg cells were co-cultured with Teff cells at a ratio of 1:3 in the anti-CD3 antibody-coated plates. Data shown are representative of three independent experiments.

T-cell-specific GLK Tg (Lck-GLK Tg) mice show normal Treg-cell development in the thymus, spleen, and peripheral blood in 5-week old mice 13; however, our previous study 9 using GLK-deficient mice suggests that GLK inhibits suppressive activity of nTreg cells. To investigate whether GLK transgene inhibits nTreg population and activity, we performed flow cytometry analysis using peripheral blood T cells of Lck-GLK Tg mice. The data showed a normal population of nTreg (CD4^+^CD25^+^Nrp-1^+^) cells in GLK Tg mice (Figure 1A). Notably, the levels of the Treg surface protein CD25 in nTreg cells were not affected by GLK transgene (Figure S1A). We also examined the suppressive function of nTreg cells from Lck-GLK Tg mice. Interestingly, GLK Tg nTreg (CD4^+^CD25^+^Nrp-1^+^) cells displayed reduction of suppressive function *in vitro* (Figure 1B). These results suggest that GLK overexpression inhibits the suppressive function but not the number of nTreg.

For the induced Treg (iTreg) cells, the intestinal immune system consists of 50-70% peripheral iTreg cells, which are rare in other secondary lymphoid systems 27. The percentage of basal CD4^+^Foxp3^+^ T cells containing both nTreg and iTreg cells in colon lamina propria (cLP) was modestly decreased in Lck-GLK Tg mice (Figure 1C). After gating CD4^+^Foxp3^+^Nrp-1^-^ group as the iTreg population, the percentage of basal iTreg cells in cLP was significantly decreased in Lck-GLK Tg mice compared to those of wild-type mice (Figure 1D). To study the iTreg activity* in vivo*, we subjected Lck-GLK Tg mice to an autoimmune disease model. Lck-GLK Tg mice were immunized with myelin oligodendrocyte glycoprotein (MOG). Exacerbated experimental autoimmune encephalomyelitis (EAE) symptoms and delayed recovery were manifested in MOG-immunized GLK Tg mice (Figure 1E), suggesting a decreased suppressive function of EAE-induced Treg cells in Lck-GLK Tg mice. Infiltrating Treg (CD4^+^Foxp3^+^) cells were decreased in the central nervous system (CNS) of Lck-GLK Tg mice in the recovery phase (Figure 1F). Immunofluorescence staining analysis also showed that infiltration of T cells was induced, whereas infiltration of Treg cells were reduced, in the brain of Lck-GLK Tg mice during EAE induction (Figure 1G). We further studied the role of GLK in iTreg differentiation *in vitro* using splenic naïve T cells from Lck-GLK Tg mice or GLK-deficient mice. The frequency of *in vitro* iTreg (CD4^+^Foxp3^+^) cells was decreased in GLK Tg T cells compared to that of wild-type T cells (Figure 1H), while the CD25 levels in the CD4^+^Foxp3^+^ gated iTreg cells were not affected by GLK transgene (Figure S1A). Consistently, Foxp3 mRNA levels were decreased in the *in vitro*-differentiated GLK transgenic T cells (Figure 1I). To study the iTreg suppressive function, *in vitro* differentiated iTreg cells were purified (Figure S1B) and co-cultured with effector T cells. *In vitro* suppression assay showed a reduction of suppressive function of GLK Tg iTreg (Figure 1J). These results suggest that GLK overexpression inhibits the populations of basal iTreg cells and inflammation-induced Treg cells, leading to enhanced susceptibility to autoimmune responses.

### IKKβ mediates the GLK-inhibited Foxp3 transcription

**Figure 2:**
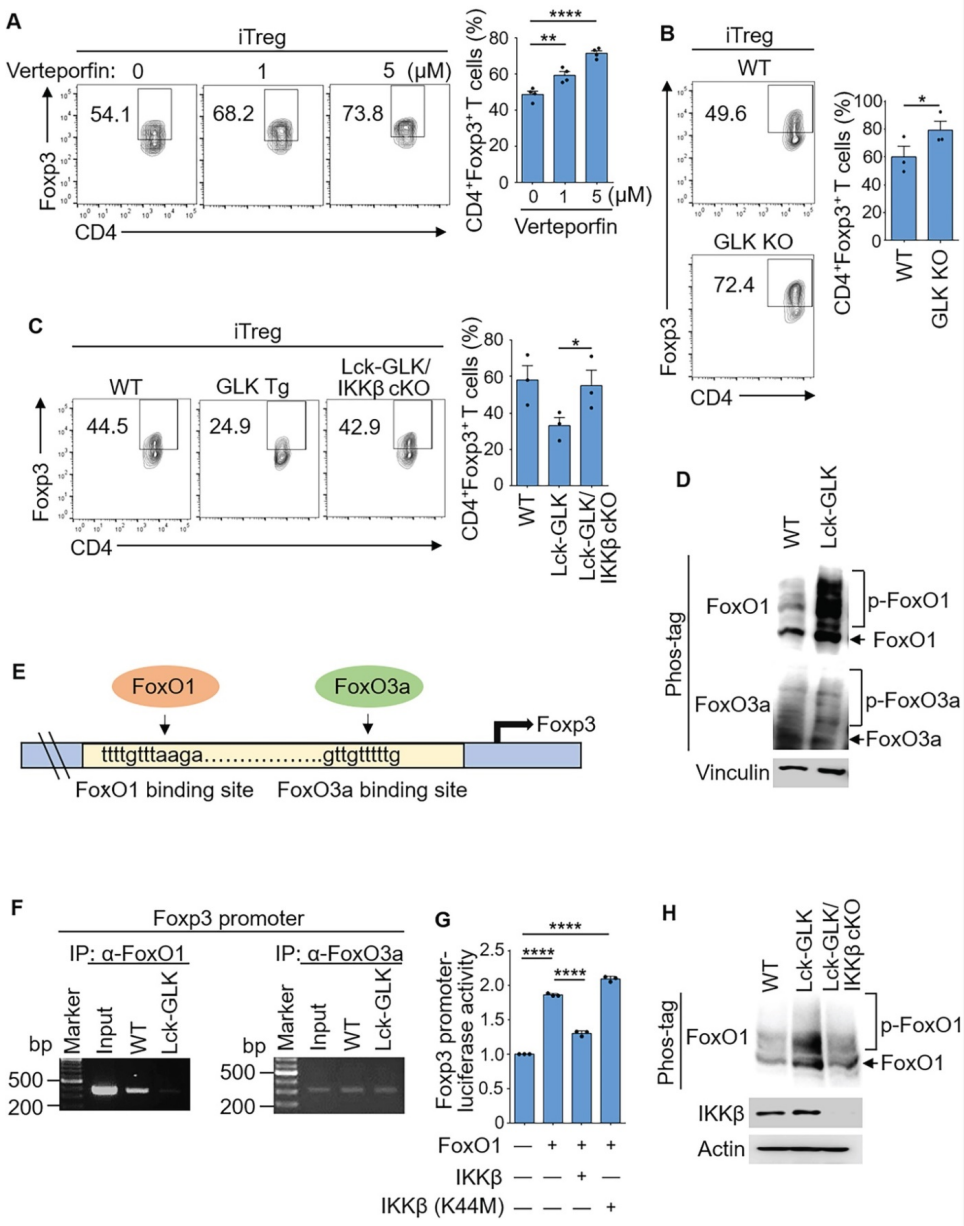
** GLK-IKKβ signaling attenuates Foxp3 promoter activity through FoxO1. (A)** Purified CD4^+^ T cells were cultured under the iTreg-differentiated condition with or without the treatment of the GLK inhibitor (1 μM or 5 μM), with a quantitative graph of CD4^+^Foxp3^+^ T cells among CD4^+^ T cells. Mean ± SEM are shown. **(B)** Flow cytometry analysis of CD4^+^Foxp3^+^ T cells among *in vitro*-differentiated iTreg cells from wild-type (WT) and GLK-deficient mice, with a quantitative graph of CD4^+^Foxp3^+^ T cells among CD4^+^ T cells. Mean ± SEM are shown.** (C)** Flow cytometry analysis of CD4^+^Foxp3^+^ T cells among *in vitro*-differentiated iTreg cells from WT, Lck-GLK Tg mice, and Lck-GLK Tg/ IKKβ cKO mice, with a quantitative graph of CD4^+^Foxp3^+^ T cells among CD4^+^ T cells. Mean ± SEM are shown. **(D)** For immunoblotting analysis of phosphorylated (p)-FoxO1, FoxO1, p-FoxO3a, FoxO3a, and vinculin in protein lysates from WT or Lck-GLK Tg T cells, Phos-tag SDS-PAGE gel was used. **(E)** The schematic diagram of FoxO1 or FoxO3a binding site on the Foxp3 promoter. Arrows indicate FoxO-binding sites. **(F)** Chromatin immunoprecipitation (ChIP)-PCR analysis of immunoprecipitated FoxO1- or FoxO3a-binding DNA fragments of the Foxp3 promoter from T cells of WT and Lck-GLK Tg mice. **(G)** The Foxp3 promoter reporter, the FoxO1 plasmid, and the plasmid encoding IKKβ or IKKβ kinase-dead (K44M) mutant were co-transfected into Jurkat T cells; the luciferase reporter activity of the Foxp3 promoter was determined. Mean ± SEM are shown.** (H)** Immunoblotting analysis of p-FoxO1, FoxO1, GLK, IKKβ, and actin in protein lysates from WT, Lck-GLK Tg, and Lck-GLK Tg/IKKβ cKO T cells using Phos-tag SDS-PAGE gel. Data shown are representative of three independent experiments.

Wild-type iTreg differentiation was enhanced by the treatment of the GLK inhibitor verteporfin (Figure 2A), which is not toxic to T cells 28. Consistent with the data from our previous report 9, the frequency of *in vitro* differentiated iTreg cells in GLK-deficient T cells was comparable to that of wild-type T cells using an optimal condition with TGF-β (10 ng/ml, Figure S1C). In contrast, GLK-deficient T cells displayed enhancement of *in vitro* iTreg differentiation under a sub-optimal condition with TGF-β (2 ng/ml, Figure 2B and Figure S1C). The different results may be due to the involvement of GLK in the TGF-β signaling axis. Taken together, these results suggest that GLK inhibits Treg differentiation. To investigate the molecular mechanism of GLK-inhibited Treg differentiation, we tested whether the GLK-downstream kinase IKKβ mediates GLK-inhibited Treg differentiation by crossing Lck-GLK transgenic mice with T-cell specific IKKβ conditional KO mice (IKKβ^f/f^;CD4-cre), resulting in Lck-GLK Tg/IKKβ cKO mice. The frequency of splenic nTreg cells in Lck-GLK Tg/IKKβ cKO mice was comparable to those of wild-type or Lck-GLK Tg mice (Figure S1D). GLK transgene-reduced Treg differentiation was reversed by IKKβ T-cell conditional knockout (Figure 2C). Consistent with enhancement of Treg differentiation by IKKβ conditional knockout, serum IL-17A levels are decreased in Lck-GLK Tg/IKKβ cKO mice compared to those of Lck-GLK Tg mice 13. These results suggest that GLK overexpression inhibits Treg differentiation through IKKβ.

Foxp3 transcription is induced by FoxO1/FoxO3a transcription factors 21, but is reduced by the phosphorylation and nuclear export of FoxO1/FoxO3a proteins 29. IKKβ directly phosphorylates and induces nuclear translocation of FoxO3a 20, 21, 24, 25, whereas it is unclear whether IKKβ can phosphorylate and regulate FoxO1. Surprisingly, the phosphorylation levels of FoxO1 but not FoxO3a in GLK transgenic T cells were enhanced compared to those of wild-type mice (Figure 2D). Next, we studied whether GLK transgene-reduced Treg differentiation is due to FoxO1/FoxO3a transcriptional repression (Figure 2E). Chromatin IP (ChIP) data showed that the binding of FoxO1 to the Foxp3 promoter was abolished in GLK-transgenic T cells; whereas the binding of FoxO3a was not affected (Figure 2F). To study whether FoxO1-induced Foxp3 transcription is attenuated by IKKβ, we tested the reporter activity of the Foxp3 promoter in Jurkat T cells. The Foxp3 promoter activity was enhanced by FoxO1, whereas IKKβ attenuated the FoxO1-induced Foxp3 promoter activity (Figure 2G). As a control, IKKβ kinase-dead (K44M) mutant did not affect the reporter activity of the Foxp3 promoter (Figure 2G). Our results also showed that FoxO1 phosphorylation was decreased in GLK Tg/IKKβ cKO T cells (Figure 2H). GLK-IKKβ signaling and TGF-β signaling might act in an antagonistic manner to regulate FoxO1-mediated Foxp3 transcription. These findings suggest that GLK-IKKβ signaling inhibits Foxp3 transcription and iTreg differentiation through FoxO1 phosphorylation.

### IKKβ directly interacts with FoxO1 and mediates FoxO1 nuclear export

**Figure 3:**
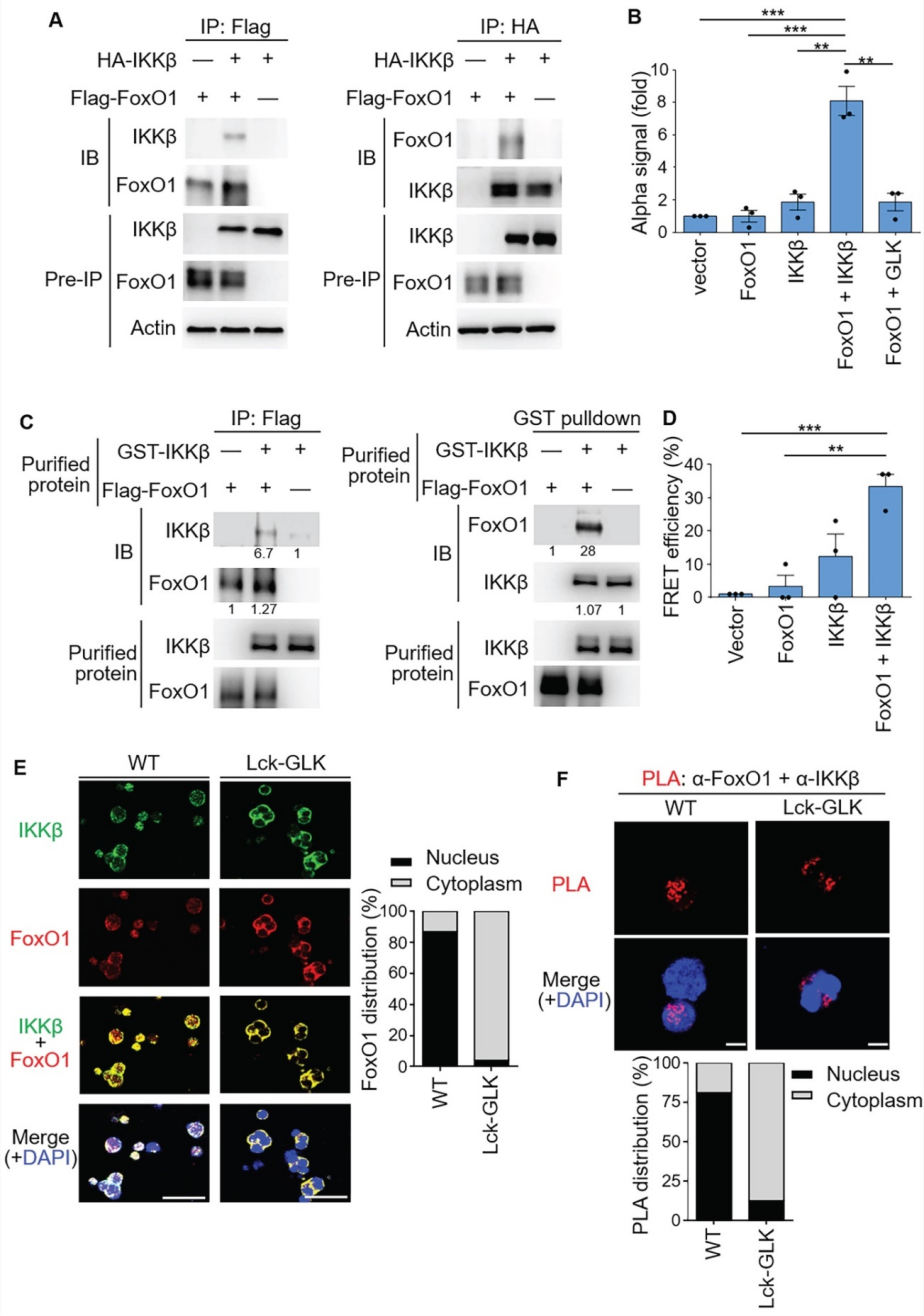
** IKKβ directly interacts with FoxO1. (A)** Co-immunoprecipitation experiments of Flag-tagged FoxO1 and HA-tagged IKKβ using lysates of HEK293T cells. IB, immunoblotting.** (B)** Amplified luminescent proximity homogeneous assay (ALPHA) analysis of the interaction between Flag-FoxO1 and either HA-IKKβ or Flag-GLK in lysates of HEK293T transfectants. Mean ± SEM are shown. **P < 0.01 and ***P < 0.001 (two-tailed student's test). **(C)** Purified Flag-tagged FoxO1 and GST-tagged IKKβ proteins were used for *in vitro* binding assay. The relative protein levels of the protein complexes determined by densitometry analysis were shown at the bottom of panels. **(D)** FRET analysis of the direct interaction between YFP-fused FoxO1 and CFP-fused IKKβ proteins in HEK293T transfectants. **P < 0.01 and ***P < 0.001 (two-tailed student's test).** (E)** Confocal microscopy analysis of intracellular localization of IKKβ and FoxO1 proteins in iTreg cells differentiated *in vitro* from T cells of wild-type (WT) and Lck-GLK Tg mice (left). Original magnification, x630; scale bars, 10 μm. FoxO1 localizations in the cytoplasm or nucleus of iTreg cells were quantified (right). **(F)** Confocal microscopy analysis of proximity ligation assay (PLA) for the interaction between endogenous FoxO1 and IKKβ proteins in iTreg differentiated from T cells of WT and Lck-GLK Tg mice (upper panel). Red dots represent direct interactions. Original magnification, x630; scale bars, 5 μm. PLA signals in the cytoplasm or nucleus of iTreg cells were quantified (lower panel). Data shown are representative of three independent experiments.

We next investigated whether IKKβ interacts with FoxO1. Co-immunoprecipitation (co-IP) assays showed an interaction between IKKβ and FoxO1 (Figure 3A). ALPHA technology/protein-protein interaction assays with specific donor/acceptor beads also confirmed the interaction between FoxO1 and IKKβ, but not between FoxO1 and GLK (Figure 3B). Purified IKKβ and FoxO1 proteins were subjected to *in vitro* binding assays; the data showed a direct interaction between these two proteins* in vitro* (Figure 3C). Furthermore, the direct interaction between IKKβ and FoxO1 in cells was demonstrated by fluorescence resonance energy transfer (FRET) analysis (Figure 3D). To examine the subcellular localization and the protein interaction, GLK transgenic iTreg were subjected to immunofluorescence microscopy and *in situ* proximity ligation assay (PLA), respectively. Immunofluorescence imaging results showed that IKKβ and FoxO1 were co-localized in the cytoplasm of GLK transgenic iTreg cells, whereas these two proteins were co-localized in the nucleus of wild-type iTreg cells (Figure 3E). The PLA signals of IKKβ-FoxO1 interaction complexes were detected in the nucleus of wild-type T cells (Figure 3F). In contrast, GLK transgene induced the nuclear export of the IKKβ-FoxO1 protein complex in iTregs (Figure 3F). These results suggest that IKKβ directly interacts with FoxO1, resulting in FoxO1 nuclear export upon GLK signaling.

### IKKβ directly phosphorylates FoxO1 at Ser319

**Figure 4:**
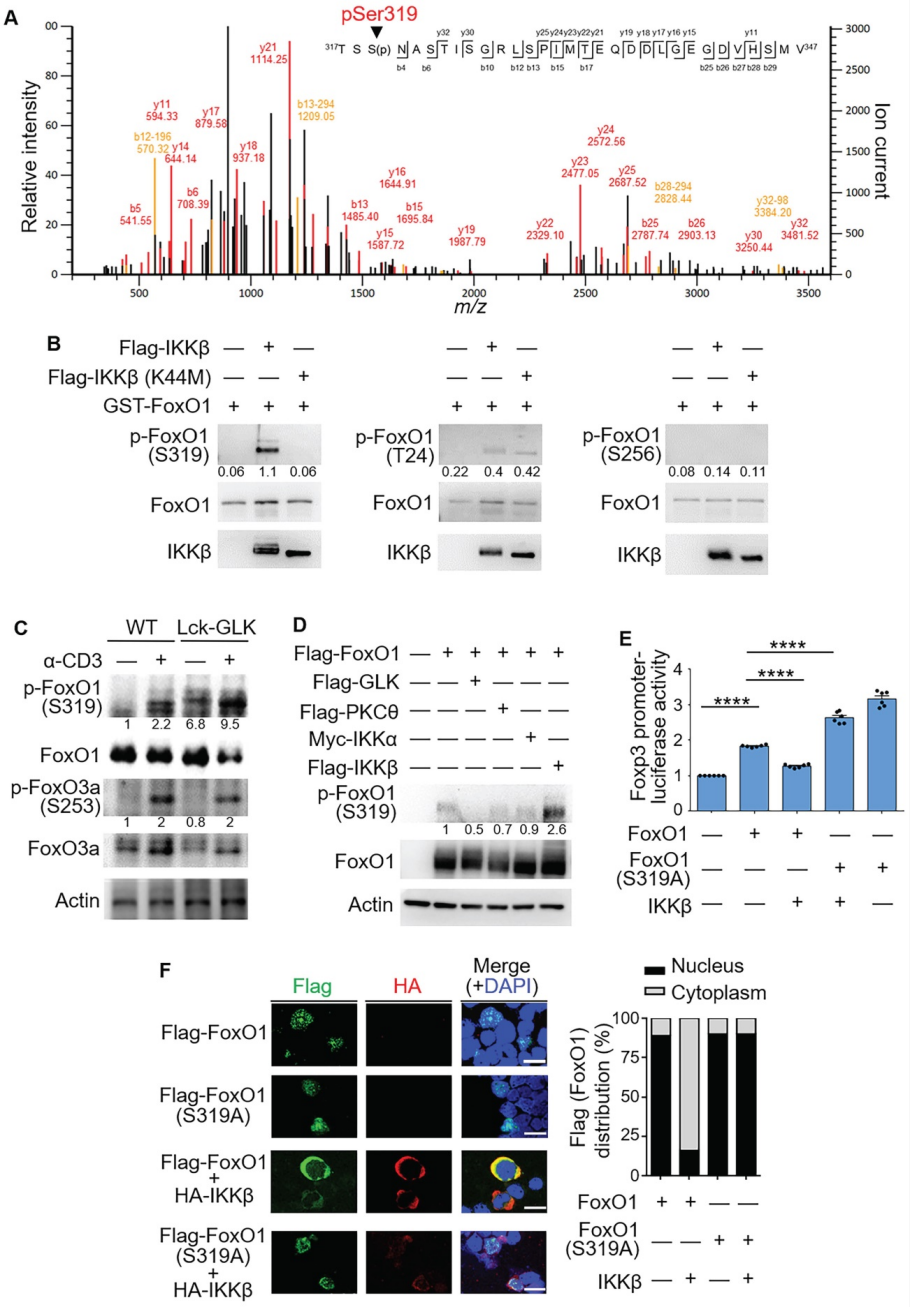
** IKKβ directly phosphorylates FoxO1 at Ser319, leading to FoxO1 nuclear export. (A)** Tandem MS (MS/MS) fragmentation spectra of the trypsin-digested FoxO1 peptides contain the phosphorylation of Ser319 with observed b/y ions. m/z, mass/charge ratio. **(B)** *In vitro* kinase assays using purified GST-tagged FoxO1 plus either Flag-tagged IKKβ or IKKβ kinase-dead (K44M) mutant proteins. Three individual phosphorylated residues of FoxO1 proteins were determined by immunoblotting analyses. The relative phosphorylation levels (phosphorylated protein level/total protein level) determined by densitometry analysis were shown at the bottom of panels. **(C)** Immunoblotting analysis of phosphorylated (p-) FoxO1 (Ser319), FoxO1, p-FoxO3a (Ser256), FoxO3a, and actin in splenic wild-type (WT) or Lck-GLK Tg T cells stimulated with or without anti-CD3 antibodies. The relative phosphorylation levels (phosphorylated protein level/total protein level) determined by densitometry analysis were shown at the bottom of panels. **(D)** Immunoblotting analysis of p-FoxO1 (Ser319), FoxO1, and actin in the lysates of HEK293T cells co-transfected with Flag-FoxO1 plus individual Flag-GLK, Flag-PKCθ, Myc-IKKα, or Flag-IKKβ plasmids. The relative phospho-Ser319-FoxO1 levels determined by densitometry analysis were shown at the bottom of the panel. **(E)** The Foxp3 promoter reporter with either FoxO1 or FoxO1 (Ser319A) plasmid plus IKKβ plasmid were co-transfected into Jurkat T cells; the luciferase reporter activity of the Foxp3 promoter was determined. Mean ± SEM are shown. **(F)** Confocal microscopy analysis of intracellular localization of HA-tagged IKKβ and Flag-tagged-FoxO1 proteins in Jurkat (J-TAg) cells co-transfected with HA-IKKβ plus either Flag-FoxO1 or Flag-FoxO1 (S319A) plasmids. Original magnification, x630; scale bars, 10 μm. Localizations of FoxO1 and FoxO1 (S319A) localizations in the cytoplasm or nucleus of Jurkat (J-TAg) cells were quantified (right panel). Data shown (B-F) are representative of three independent experiments.

FoxO1 phosphorylation determines the transcriptional activity and cellular localization of FoxO1 29. Our results showed that GLK-IKKβ signaling triggered the nuclear export of FoxO1. To identify the specific IKKβ-targeted FoxO1 phosphorylation site that regulates the nuclear export of FoxO1, we purified *in vitro* phosphorylated Flag-tagged FoxO1, followed by mass spectrometry (MS) analyses. Ser319, but not Thr24, was identified as the FoxO1 phosphorylation site by IKKβ (Figure 4A). Moreover, we performed *in vitro* kinase assay using purified GST-tagged FoxO1 with either Flag-tagged IKKβ or Flag-tagged IKKβ kinase-dead (K44M) mutant proteins. IKKβ drastically induced FoxO1 phosphorylation at Ser319 (Figure 4B), while IKKβ slightly induced Thr24 phosphorylation of FoxO1 (Figure 4B). In contrast, FoxO1 Ser319 phosphorylation was not induced by IKKβ kinase-dead (K44M) mutant (Figure 4B), suggesting that IKKβ-induced FoxO1 Ser319 phosphorylation is dependent on IKKβ kinase activity. Nevertheless, we could not rule out the possibility that GLK-IKKβ signaling stimulates an additional phosphorylation site(s) of FoxO1. Collectively, our data suggest that active IKKβ mainly phosphorylates FoxO1 at Ser319 residue.

In GLK transgenic T cells, the phosphorylation of Ser319 on FoxO1 was further enhanced compared to those of wild-type T cells upon anti-CD3 stimulation (Figure 4C), while AKT-mediated FoxO3a Ser253 phosphorylation 20 was not further enhanced by GLK transgene in TCR-stimulated T cells (Figure 4C). Moreover, Ser319 phosphorylation levels on FoxO1 proteins in HEK293T cells were enhanced by overexpression of IKKβ but not GLK, PKCθ, or IKKα (Figure 4D). To study whether IKKβ-induced Ser319 phosphorylation on FoxO1 is responsible for inhibition of FoxO1-mediated Foxp3 transcription, we generated the phospho-deficient FoxO1 (S319A) mutant. As expected, the IKKβ-inhibited FoxO1 transcriptional activity was recovered by the phospho-deficient FoxO1 (S319A) mutation (Figure 4E). IKKβ overexpression caused predominantly cytoplasmic localization of FoxO1 in transfected Jurkat (J-TAg) cells (Figure 4F). Consistently, the FoxO1 mutant (S319A) was retained in the nucleus in IKKβ-transfected Jurkat (J-TAg) cells (Figure 4F). Thus, the data suggest that IKKβ phosphorylates FoxO1 at Ser319, resulting in FoxO1 nuclear export and Foxp3 transcriptional repression.

### GLK directly phosphorylates IKKβ at Ser733 and subsequently induces IKKβ-mediated FoxO1 phosphorylation

**Figure 5:**
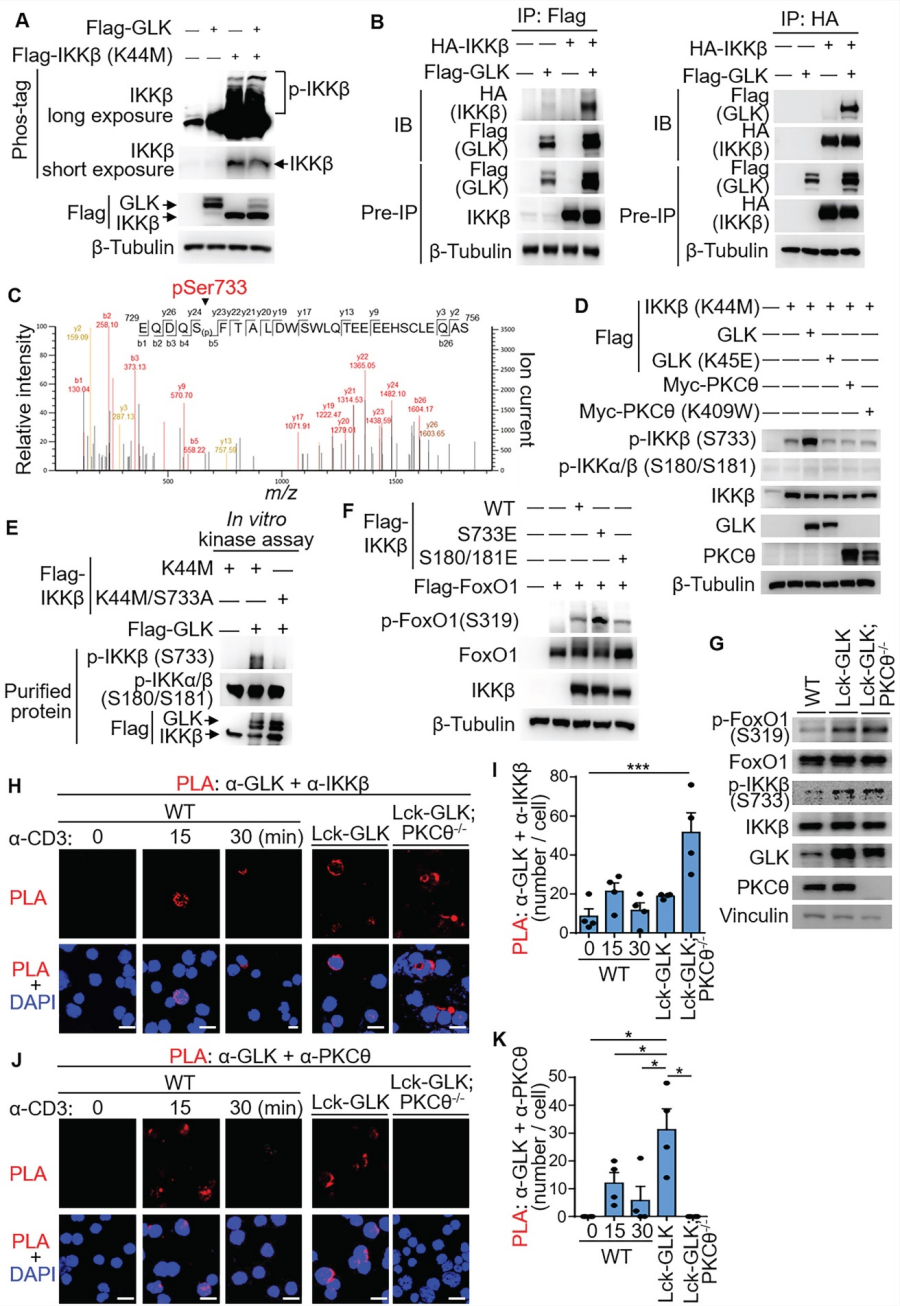
** GLK directly phosphorylates IKKβ at Ser733, leading to IKKβ-induced FoxO1 Ser319 phosphorylation. (A)** For immunoblotting analysis of phosphorylated (p-) IKKβ, IKKβ proteins in protein lysates from HEK293T co-transfected with Flag-GLK and Flag-IKKβ kinase-dead (K44M) plasmids, Phos-tag SDS-PAGE gel was used. **(B)** Co-immunoprecipitation experiments of Flag-tagged GLK and HA-tagged IKKβ using lysates of transfected HEK293T cells. IB, immunoblotting. **(C)** Tandem MS (MS/MS) fragmentation spectra of the trypsin-digested peptides of IKKβ contain the phosphorylation of Ser733 with observed b/y ions. m/z, mass/charge ratio. Flag-tagged-IKKβ kinase-dead (K44M) mutant proteins were immunoprecipitated from lysates of HEK293T cells co-transfected with Flag-IKKβ kinase-dead (K44M) mutant and Flag-GLK plasmids. **(D)** Immunoblotting analyses of p-IKKβ (S733), p-IKKα/β (S180/S181), IKKβ, GLK, and PKCθ in HEK293T cells. Cells were co-transfected with Flag-IKKβ kinase-dead (K44M) mutant plus individual Flag-GLK, Flag-GLK kinase-dead (K45E) mutant, Myc-PKCθ, or Myc-PKCθ kinase-dead (K409W) mutant plasmids. **(E)** *In vitro* kinase assays of Flag-tagged GLK with either Flag-tagged IKKβ kinase-dead (K44M) mutant or Flag-tagged IKKβ phospho-deficient (K44M/S733A) mutant proteins immunopurified from individual HEK293T transfectants. **(F)** Immunoblotting analyses of p-FoxO1 (Ser319) and FoxO1 proteins in HEK293T cells co-transfected with Flag-FoxO1 plus individual Flag-IKKβ, phosphomimetic Flag-IKKβ (S733E) mutant, or phosphomimetic Flag-IKKβ (S180E/S181E) mutant plasmids. **(G)** Immunoblotting analyses of the endogenous p-FoxO1 (Ser319), FoxO1, p-IKKβ (Ser733), IKKβ, GLK, and PKCθ proteins in primary splenic T cells from wild-type (WT), Lck-GLK Tg, and Lck-GLK Tg/PKCθ KO mice. **(H)** Confocal microscopy analysis of proximity ligation assay (PLA) for the interaction between endogenous GLK and IKKβ proteins in T cells from WT, Lck-GLK Tg, and Lck-GLK Tg/PKCθ KO mice. T cells were stimulated with anti-CD3 antibodies plus streptavidin (3 μg/ml). Red dots represent direct interactions. Original magnification, x630; scale bars, 10 μm. **(I)** Quantification of the PLA signals (H) in each cell is shown. **(J)** Confocal microscopy analysis of PLA for the interaction between endogenous GLK and PKCθ proteins in T cells from WT, Lck-GLK Tg, and Lck-GLK Tg/PKCθ KO mice. T cells were stimulated with anti-CD3 antibodies plus streptavidin (3 μg/ml). Red dots represent direct interactions. Original magnification, x630; scale bars, 10 μm. **(K)** Quantification of the PLA signals (J) in each cell is shown. Data shown (A, B, D-K) are representative of three independent experiments.

The IKKβ-induced interaction between AhR and RORγt is detectable in the PKCθ KO T cells indicating that GLK can stimulate IKKβ in the absence of PKCθ 13. To study whether GLK is an upstream kinase of IKKβ, we used Phos-tag SDS-PAGE gel analysis using HEK293T lysates from individually transfected cells. To avoid autophosphorylation of IKKβ, IKKβ kinase-dead (K44M) mutant was used as the substrate. Phosphorylation levels of IKKβ kinase-dead (K44M) mutant were enhanced by GLK (Figure 5A). Co-immunoprecipitation data showed that IKKβ interacted with GLK (Figure 5B). To test whether GLK directly phosphorylates IKKβ, we performed *in vitro* kinase assays using purified GLK and IKKβ proteins from individually transfected cells. Interestingly, serine phosphorylation levels of IKKβ was induced by GLK *in vitro* (Figure S2A). To identify the GLK-induced IKKβ phosphorylation site, GLK-phosphorylated IKKβ kinase-dead (K44M) mutant was subjected to mass spectrometry analyses. Surprisingly, Ser733 was identified as the IKKβ phosphorylation site by GLK (Figure 5C). The phospho-Ser733-IKKβ levels of wild-type IKKβ and IKKβ kinase-dead (K44M) mutant proteins were increased by GLK overexpression in a dose-dependent manner (Figure S2B-C). To rule out the possibility that PKCθ induces IKKβ Ser733 phosphorylation, we co-transfected IKKβ kinase-dead (K44M) mutant plus individual GLK, GLK kinase-dead (K45E) mutant, or PKCθ plasmids into HEK293T cells. The data showed that the kinase GLK, but not PKCθ, drastically induced IKKβ Ser733 phosphorylation (Figure 5D). Flag-tagged IKKβ kinase-dead (K44M) mutant but not phospho-deficient IKKβ (K44M/S733A) mutant was phosphorylated by purified GLK at Ser733 (Figure 5E); the data also confirmed the specificity of phospho-Ser733-IKKβ antibody. Collectively, these results suggest that GLK overexpression directly induces IKKβ Ser733 phosphorylation.

Next, we investigated whether phospho-IKKβ Ser733 stimulates FoxO1 Ser319 phosphorylation. Overexpression of phosphomimetic IKKβ (S733E) mutant in HEK293T cells enhanced FoxO1 Ser319 phosphorylation (Figure 5F). Overexpression of IKKβ induces FoxO3a Ser644 phosphorylation in cancer cells 24. Interestingly, phosphomimetic IKKβ (S733E) mutant did not induce FoxO3a phosphorylation at Ser644 (Figure S3), suggesting that Ser733-phosphorylated IKKβ selectively induces phosphorylation of FoxO1 instead of FoxO3a. For murine primary T cells, FoxO1 Ser319 phosphorylation and IKKβ Ser733 phosphorylation were both induced in the GLK transgenic T cells (Figure 5G). To confirm the direct interaction between GLK with either IKKβ or PKCθ in cells, we performed *in situ* PLA using probes corresponding to either GLK plus IKKβ or GLK plus PKCθ. The interaction between GLK and IKKβ was readily detected in Lck-GLK transgenic T cells or Lck-GLK/PKCθ^-/-^ T cells, but not in wild-type T cells (Figure 5H-I). Interestingly, more PLA signals were detected in Lck-GLK PKCθ^-/-^ T cells (Figure 5H and Figure S4). It is possible that more free GLK proteins are available to interact with IKKβ in the absence of PKCθ in Lck-GLK PKCθ^-/-^ T cells. As a control, the interaction between GLK and PKCθ was induced in TCR-stimulated or GLK transgenic T cells, but not in Lck-GLK/PKCθ^-/-^ T cells (Figure 5J-K). These results indicate that GLK overexpression or GLK transgene indirectly induces FoxO1 Ser319 phosphorylation via stimulating IKKβ Ser733 phosphorylation.

### GLK transgene decreases Foxp3 transcripts through FoxO1

**Figure 6:**
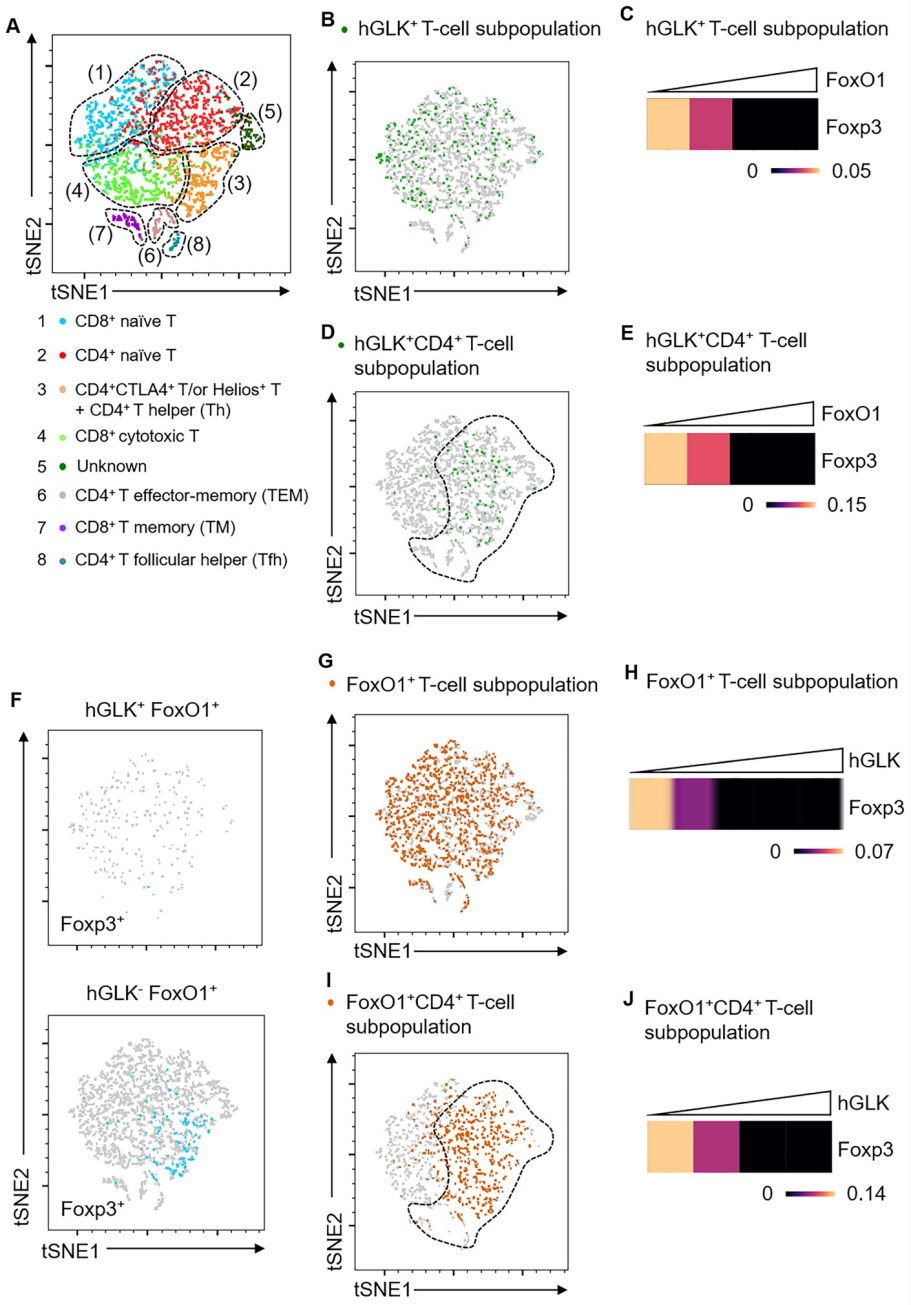
** Foxp3 mRNA levels are inversely correlated with FoxO1 mRNA levels in GLK transgenic T cells. (A)** Two-dimensional tSNE plot of T cell single-cell transcriptomes with different areas identified by clustering. The T cell subset in each area is indicated. **(B)** tSNE plot showing GLK^+^ T-cell subpopulation (6.86%) in total T cells. **(C)** Heatmap showing Foxp3 transcripts in GLK^+^ T-cell subpopulation. **(D)** tSNE plot showing GLK^+^CD4^+^ T-cell subpopulation (4.96%) in total T cells. **(E)** Heatmap showing Foxp3 transcripts in GLK^+^CD4^+^ T-cell subpopulation. **(F)** tSNE plots showing 1.14% of Foxp3^+^ T cells in GLK^+^FoxO1^+^ T cells (upper panel) or 3.9% of Foxp3^+^ T cells in GLK^-^FoxO1^+^ T cells (lower panel). **(G)** tSNE plot showing FoxO1^+^ T-cell subpopulation (36.8%) in total T cells. **(H)** Heatmap showing Foxp3 downregulation in FoxO1^+^ T-cell subpopulation with concomitantly elevated GLK expression. **(I)** tSNE plot showing FoxO1^+^ T-cell subpopulation (36.8%) in CD4^+^ T cells.** (J)** Heatmap showing Foxp3 expression in FoxO1^+^CD4^+^ T-cell subpopulation with concomitantly elevated GLK expression.

GLK-IKKβ signaling inhibited Treg differentiation and suppressive function; thus, we studied whether GLK signaling regulates Treg transcriptional profiles. To study the GLK signaling-dependent transcriptomes, we performed scRNA-seq using splenic T cells from wild-type and Lck-GLK Tg mice. Dimensionality reduction/clustering analysis by tSNE showed eight distinct clusters of T cells (Figure 6A). In GLK^+^ T cells, GLK transgene gradually decreased Foxp3 transcripts with the concomitant increase of FoxO1 expression (Figure 6B-C). Consistently, GLK transgenic CD4^+^ T cells showed reduction of Foxp3 expression correlates with the presence of FoxO1 transcripts in a dose-dependent manner (Figure 6D-E). Regardless of FoxO1 levels, Foxp3 expression was not detectable in GLK^+^CD8^+^ T cells (Figure S5). Foxp3 transcripts were more abundant in GLK^-^FoxO1^+^ T cells compared to those of GLK^+^FoxO1^+^ T cells (Figure 6F). Moreover, Foxp3 expression showed no correlation with FoxO1 transcripts in GLK^-^ T cells (Figure S6). GLK overexpression also inhibited Foxp3 expression in FoxO1^+^ T cells in a dose-dependent manner (Figure 6G-H). In FoxO1^+^CD4^+^ T cells, Foxp3 transcripts also were gradually decreased with concomitantly increased GLK expression (Figure 6I-J). Foxp3 expression levels in total T cells, FoxO1^+^ T-cell subpopulation, and FoxO1^+^CD8^+^ T-cell subpopulation were comparable between wild-type and Lck-GLK Tg mice (Figure S7-Figure S9). In contrast, Foxp3 transcripts were decreased in FoxO1^+^CD4^+^ T-cell subpopulation in Lck-GLK Tg mice (Figure S10A-C). These data support a critical role of GLK signaling in FoxO1-mediated attenuation of Foxp3 transcription. Furthermore, Gene Ontology (GO) enrichment analysis showed that differentially expressed genes (DEGs) in total T cells (Figure S8B) were mainly involved in the cellular metabolic pathway, cellular responses to external stimuli, and AP1 signaling transduction (Figure S11 and Table S1). In FoxO1^+^ T cells (Figure S8A), DEGs also showed similar gene alteration profiles compared to those of total T cells (Figure S8B and Table S2). Taken together, these results suggest that Foxp3 expression is attenuated by GLK-IKKβ-signaling-mediated FoxO1 phosphorylation/nuclear export, leading to inhibition of Treg differentiation and suppressive function.

## Discussion

A major finding of our study is that the GLK-IKKβ axis attenuated FoxO1-induced Foxp3 transcription. FoxO1 is crucial for the suppressive function in Treg cells 26. Our data showed that IKKβ-induced FoxO1 Ser319 phosphorylation in GLK-overexpressing T cells, resulting in FoxO1 nuclear export and Foxp3 downregulation. Consistently, scRNA-seq data showed that Foxp3 mRNA levels were inversely correlated with FoxO1 mRNA levels in GLK transgene-positive T cell subpopulation. T-cell-specific GLK transgenic mice displayed normal nTreg populations in the peripheral blood and spleen. Nevertheless, the suppressive function of nTreg cell was still suppressed by GLK overexpression. Moreover, GLK overexpression in T cells inhibited both basal iTreg and disease-induced Treg populations, as well as iTreg suppressive activity. Collectively, our findings suggest that GLK-IKKβ-FoxO1 signaling in T cells suppresses regulatory T cells, leading to enhanced susceptibility to autoimmune diseases.

Another key finding in this report is that GLK directly interacted with and phosphorylated IKKβ at Ser733 in a PKCθ-independent manner, resulting in selective phosphorylation of FoxO1 but bot FoxO3a. Subsequently, Ser733-phosphorylated IKKβ inhibited the FoxO1 transcriptional activity on the Foxp3 promoter by inducing FoxO1 phosphorylation and nuclear export. Upon TNF-α stimulation, phosphorylation of 10 serine residues (including Ser733) at the C-terminal region of IKKβ, mimicked by a mutant with 10 serine-to-glutamic acid residues, results in the inhibition of IKKβ kinase activity *in vitro* 30. It is likely that phosphorylation of one or more of the other 9 serine residues, but not Ser733, on IKKβ is responsible for IKKβ inactivation. In addition, IKKβ Ser181 is phosphorylated in response to proinflammatory cytokines, including TNF-α and IL-1β 30. In GLK-overexpressing T cells, IKKβ stimulates IL-17A transcription by phosphorylating RORγt 13. These findings suggest that the phosphorylation of different IKKβ residues controls different functions of IKKβ by regulating its kinase activity 31 and/or interaction with its targets. Thus, GLK overexpression in T cells induces IKKβ activation, leading to both induction of IL-17A production and inhibition of Foxp3 transcription.

IKKβ plays complex roles in Treg development and activity. Treg cell development in the thymus is reduced in T-cell-specific IKKβ conditional knockout mice 32. In contrast, Treg-specific IKKβ conditional knockout mice display normal Treg development and enhanced Treg function, but show decreased Treg homeostasis in the spleen 33. Our findings showed that GLK overexpression in T cells induced IKKβ activation but does not affect thymic Treg development 13 or splenic nTreg population (this study). It is possible that the GLK-IKKβ-FoxO1 signaling pathway plays a less important role in thymic Treg development and peripheral homeostasis. In contrast, GLK-IKKβ-FoxO1 signaling in T cells inhibited differentiation of iTreg and activity of both nTreg and iTreg. Conversely, loss of IKKβ in GLK transgenic T cells or inhibition of GLK by a GLK inhibitor in wild-type T cells resulted in induction of Treg differentiation. Consistently, blocking NF-κB signaling enhances human Treg suppressive function 34. These findings suggest that GLK-IKKβ-FoxO1 signaling in T cells suppresses Treg differentiation and activity, leading to the blocking of Treg-mediated immune suppression.

**Figure 7:**
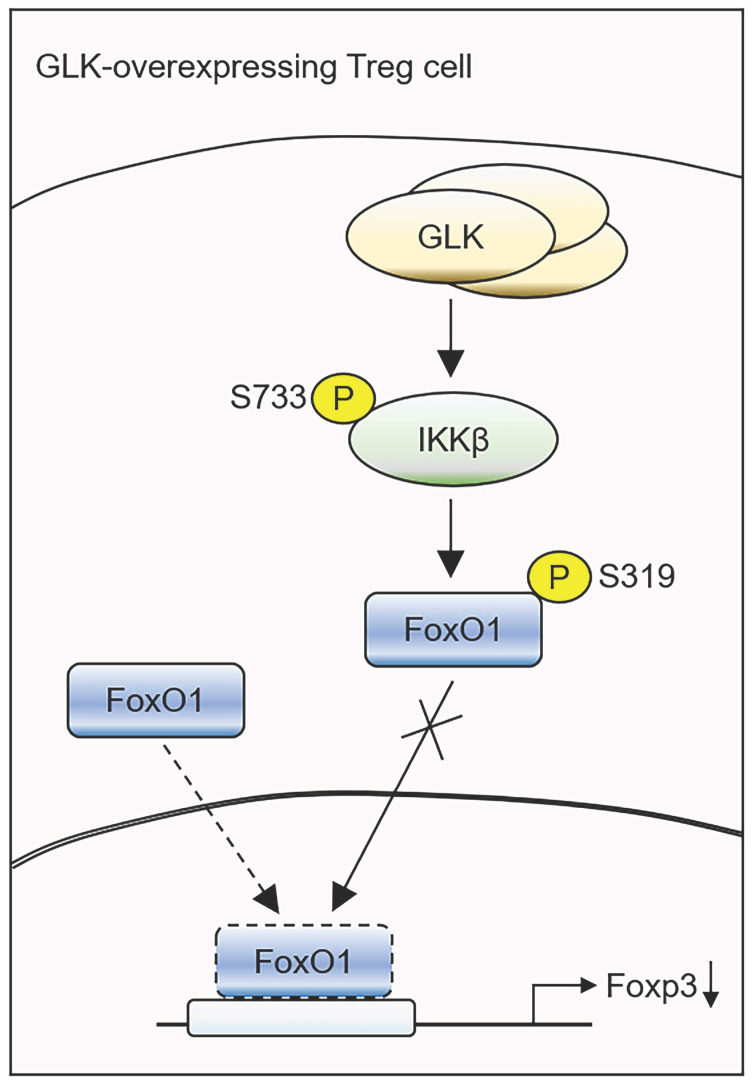
** Schematic model of GLK overexpression-induced attenuation of Foxp3 transcription.** GLK overexpression in Treg cells phosphorylates IKKβ at Ser733 residue in a PKCθ-independent manner. The transcription factor FoxO1 binds to the Foxp3 promoter and promotes Foxp3 transcription in normal Treg cells. The GLK-phosphorylated IKKβ interacts with and induces FoxO1 Ser319 phosphorylation, leading to FoxO1 nuclear export and Foxp3 downregulation. Thus, GLK-IKKβ-FoxO1 signaling attenuates differentiation and function of Treg cells.

Taken together, our study reveals a critical mechanism of GLK-IKKβ signaling-inhibited Foxp3 transcription in T cells (Figure 7). GLK overexpression-induced IKKβ Ser733 phosphorylation and subsequent IKKβ-mediated FoxO1 Ser319 phosphorylation, resulting in FoxO1 nuclear export and Foxp3 downregulation. Thus, GLK is a promising therapeutic target by both blocking IL-17A-mediated autoimmune diseases 10 and enhancing Treg-mediated immune suppression. It would be valuable to evaluate the clinical application of the GLK inhibitor in the GLK-associated autoimmune diseases, such as SLE and RA 9-11, 14, 35.

## Materials and Methods

### Mice

All mice were bred under specific pathogen-free facility at National Health Research Institutes (NHRI) and maintained according to institutional guidelines of the Institutional Animal Care and Use Committee of NHRI. All mouse lines were purchased and backcrossed as described previously 13. All animal experiments were performed under the animal protocol approved by Institutional Animal Care and Use Committee of NHRI. Sex matched, 8- to 15-week-old mice were used in this study. Floxed IKKβ mice (EMMA 001921) were purchased from the Jackson Laboratory (JAX). The data presented in this study were performed on sex-matched, 8- to 15-week-old littermates. All mice used in this study were maintained in temperature-controlled and pathogen-free cages. Lck-GLK Tg mice in C57BL/6 background were generated as described previously 13. In Lck-GLK Tg mice, a full-length human GLK coding sequence was placed downstream of the proximal Lck promoter 36.

### Cells

HEK239T cells and human Jurkat T leukemia cells were obtained from American Type Culture Collection (ATCC). Human Jurkat TAg (J-TAg) cell clone was derived from the Jurkat T cell line by stably transfecting with the simian virus 40 large T antigen. HEK293T cells were cultured and maintained in Dulbecco's modified Eagle's medium (DMEM, Thermo Fisher Scientific) supplemented with 10% fetal bovine serum (FBS, Corning Incorporated) plus penicillin (10 U/ml, Thermo Fisher Scientific) and streptomycin (10 μg/ml, Thermo Fisher Scientific). Jurkat T cells and Jurkat (J-TAg) cells were maintained in RPMI-1640 (Thermo Fisher Scientific) supplemented with 10% FBS plus penicillin (10 U/ml, Thermo Fisher Scientific) and streptomycin (10 μg/ml, Thermo Fisher Scientific). All cell lines were confirmed to be free from mycoplasma contamination.

### Antibodies, plasmids, and recombinant proteins

Anti-Flag (clone M2, #F1804), anti-HA (clone 12CA5, #11583816001), anti-phospho-serine (clone 4A4, #05-1000), anti-vinculin (clone 7F9, #MAB3574), and anti-FoxO1 (Thr24) antibodies (#07-2126) were purchased from MilliporeSigma. Antibody specific for β-tubulin (clone BT7R, #MA5-16308) was purchased from Thermo Fisher Scientific. Anti-phospho-FoxO1 (Ser319) antibody (#GTX50196) was purchased from GeneTex. Anti-actin (clone E814), anti-PKCθ (clone EPR1487(2), #ab110728), and anti-phospho-IKKβ (Ser180/181) antibodies (#ab55341) were purchased from Abcam. Anti-phospho-threonine/tyrosine (#9381), anti-phospho-FoxO1 (Ser256) (#PA5-17907), anti-phospho-FoxO1 (Ser319) (#2486), anti-FoxO1 (clone C29H4, #2880), anti-IKKβ (clone L570, #2678), anti-FoxO3a (clone 75D8, #2497), and anti-phospho-FoxO3a (Ser253) (#9466) antibodies were purchased from Cell Signaling Technology. Anti-phospho-IKKβ (Ser733) antibody (#LS-C157829) was purchased from LSBio. Anti-GLK antibody (clone C3) was generated as described previously 13. The GLK inhibitor verteporfin 28 was purchased from MilliporeSigma. Construction of the expression plasmids for wild-type IKKβ, the IKKβ kinase-dead (K44M) mutant, the phosphomimetic IKKβ (S180/181E) mutant, the GLK kinase-dead (K45E) mutant, and the PKCθ kinase-dead (K409W) mutant were described previously 13. The phospho-deficient IKKβ (K44M/S733A) mutant and the phosphomimetic IKKβ (S733E) mutant were generated by site-directed mutagenesis. Flag-tagged FoxO1-S319A mutant was generated by site-directed mutagenesis. A point mutation of Ser319 residue was substituted with alanine residue to generate the phospho-deficient FoxO1 (S319A) mutant. YFP-fused FoxO1 or Flag-tagged phospho-deficient FoxO1 (S319A) mutant plasmids were constructed by subcloning FoxO1 PCR products into individual pCMV6-AC-YFP (OriGene Technologies), pCMV6-AC-Flag (OriGene Technologies), and pGEM-4T vectors (Promega). For *in vitro* binding assay, purified FoxO1 proteins were isolated from *Escherichia coli* (BL21) and then subjected to GST pulldown assay.

### Isolation of lamina propria lymphocytes from the colon

Large intestines were collected from mice. Large intestines were treated sequentially with 2 mM dithiothreitol (DTT, MilliporeSigma) solution, 5 mM EDTA (Thermo Fisher Scientific) in HBSS solution, and then 100 U/ml collagenase Type I (MilliporeSigma) solution. Colon lamina propria (cLP) lymphocytes were collected and enriched by 44:67% Percoll (Cytiva) gradient.

### Induction of myelin oligodendrocyte peptide-induced EAE

These experiments were performed as described preciously 9.

### Isolation of lymphocytes from brain and spinal cord

The brain and spinal cord of mice were harvested and incubated with RPMI-1640 media in the gentleMACS^TM^ C tubes (Miltenyi Biotec), followed by homogenization using gentleMACS^TM^ dissociator (Miltenyi Biotec). Lymphocytes were separated and enriched by 30:70% Percoll (Cytiva) gradient.

### Purification of murine T cells

Peripheral blood, the spleen, inguinal lymph nodes, and mesenteric lymph nodes were harvested from mice for purification of primary T cells. Peripheral blood samples were collected biweekly from wild-type and Lck-GLK Tg mice. Antibody cocktails against CD11b, B220, CD49b, CD235, TER-119, Nrp-1, and CD8 (magnetic cell separation kit, Miltenyi Biotec) were used to deplete leukocytes from the organ and tissues, resulting in purified CD4^+^ T cells.

### Generation of induced regulatory T cells

Murine induced Treg (iTreg) cells were generated from splenic naïve T (CD4^+^CD62L^+^) cells. Murine iTreg cells were generated *in vitro* in RPMI-1640 (Thermo Fisher Scientific) supplemented with TGF-β recombinant proteins (2 ng/ml, R&D Systems), IL-2 recombinant proteins (10 ng/ml, R&D Systems), anti-IFN-γ (2.5 μg/ml, BioLegend), and anti-IL-4 (2.5 μg/ml, BioLegend) antibodies, and were cultured in the anti-CD3 (2 μg/ml, BD Biosciences) plus anti-CD28 (3 μg/ml, BD Biosciences) antibody-coated 48-well plate.

### *In vitro* nTreg and iTreg suppression assays

Murine CD4^+^ T cells were negatively selected from the cells of the spleen and lymph nodes. For the preparation of effector T cells, regulatory T cells were depleted from the CD4^+^ T cells using biotin-conjugated anti-mouse CD25 antibody (clone PC61, BioLegend) by MACS magnetic-bead separation (Miltenyi Biotec). For purification of nTreg cells, nTreg cells were isolated from CD4^+^ T cells using biotin-conjugated anti-mouse Nrp-1 antibody (clone 3E12, BioLegend) by MACS magnetic-bead separation (Miltenyi Biotec). For purification of the *in vitro* differentiated iTreg cells, iTreg cells were isolated from the *in vitro* differentiated CD4 cells using biotin-conjugated anti-mouse CD25 antibody (clone PC61, BioLegend) by MACS magnetic-bead separation (Miltenyi Biotec). The purity of iTreg (CD4^+^Foxp3^+^) cells was 90% (Figure S1B). For determination of effector T cell proliferation, effector T cells were labeled with 1 μM CFSE (Thermo Fisher Scientific). 5
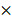
10^5^ CFSE-labelled effector T cells were co-cultured with 1.66
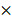
10^5^ Treg cells for 4 days under the stimulation of anti-CD3 antibody (1 μg/ml, BD Biosciences) coated on 96-well round-bottom plates.

### Flow cytometry analysis

Prediluted antibodies were prepared for staining of cell surface markers. For intracellular staining, cells were permeabilized in Foxp3 Fix/Perm solution (BioLegend) and incubated with anti-Foxp3 antibodies diluted in Foxp3 Perm buffer (BioLegend). The antibodies used: anti-CD3-PerCP (clone 145-2C11, BioLegend), anti-CD4-Pacific blue (clone RM4-5, BioLegend), anti-Foxp3-Alexa Fluor 488 (clone FJK-16s, Thermo Fisher Scientific), and anti-Nrp-1-APC (clone 3DS304M, Thermo Fisher Scientific). FACSCanto II (BD Biosciences) and FlowJo software (BD Biosciences) were used to collect and analyze the data.

### Chromatin immunoprecipitation (ChIP) assay

The sample preparation and chromatin immunoprecipitation (ChIP) assays were performed as described previously 13. PCR purification kits (GE Healthcare) were used to purify immune-enriched DNA fragments immunoprecipitated by anti-FoxO1 (Cell Signaling Technology) or anti-FoxO3a antibodies (Cell Signaling Technology). The DNA fragments were prepared for PCR amplification of 35 cycles. PCR primers were as follows: FoxO1-binding site, 5'- GCT TCA GAT CCC TTC TTC TGT TCA ACC-3' (forward) and 5'- GAG TGT GTG TGC TGA TAA TTG CAG G-3' (reverse); FoxO3a-binding site, 5'- CCT GCA ATT ATC AGC ACA CAC ACT C-3' (forward) and 5'- GGT ATT AGT TTC CAA AGT CCT TAC CTG GAG-3' (reverse).

### Transient transfection and luciferase reporter assay

The murine Foxp3 promoter (region -500/+100 bp relative to the Foxp3 transcription start site) was subcloned into pGL3 basic vector (Promega) containing firefly luciferase. The Foxp3-promoter/pGL3 reporter plasmid was co-transfected with renilla luciferase control plasmid (pRL-TK), FoxO1, FoxO1 (S319A), IKKβ, or IKKβ kinase-dead (K44M)-encoding plasmids alone or in different combinations into Jurkat T cells using Neon^®^ transfection system (Thermo Fisher Scientific). After 24 to 48 h, cells were lysed using lysis buffer (#E1910, Promega). Results represent the mean ratios of the firefly luciferase activity to the renilla luciferase activity.

### Immunoprecipitation, GST pulldown, and immunoblotting analyses

Immunoprecipitation, GST pulldown assays and immunoblotting analyses were performed as described previously 13.

### Phos-tag immunoblotting

To determine phospho-FoxO1, phospho-FoxO3a, and phospho-IKKβ protein levels, Phos-tag SDS-PAGE 37 containing 50 mM Phos-tag acrylamide (Fujifilm) was used. Briefly, phosphorylated proteins and non-phosphorylated proteins were separated by Phos-tag SDS-PAGE. Phos-tag SDS-PAGE gel contained divalent Mn^2+^ ions, which trapped the phosphorylated proteins (such as p-FoxO1/p-FoxO3a/p-IKKβ) and increased their molecular weights. After electrophoresis, the Phos-tag gel was soaked in blotting buffer containing 1 mM EDTA (MilliporeSigma). The phosphorylated proteins and non-phosphorylated proteins on the Phos-tag gel were then transferred onto polyvinylidene difluoride membranes. The membranes were blocked with 5% BSA in Tris-buffered saline with 0.1% Tween^®^ 20 (TBST) for 2 h, and then immunoblotted with anti-FoxO1, anti-FoxO3a, and anti-IKKβ antibodies to distinguish phosphorylated proteins from non-phosphorylated proteins.

### Amplified luminescent proximity homogeneous assay (ALPHA)

ALPHA protein-protein interaction assays 38 were performed according to the manufacturer's protocol. When the pairs of Flag-tagged FoxO1 and HA-tagged IKKβ proteins are within 200 nm, luminescent signals can be detected from AlphaLISA beads (PerkinElmer Life science) by an EnVision 2104 Multilabel Plate Reader (PerkinElmer Life science).

### Fluorescence resonance energy transfer (FRET) assays

YFP-FoxO1 and CFP-IKKβ plasmids were co-transfected into HEK293T cells. When the pairs of YFP-fused FoxO1 and CFP-fused IKKβ proteins are within 8 nm, YFP signals will be stimulated by CFP signals. These fluorescence signals were detected by an EnVision 2104 Multilabel Plate Reader (PerkinElmer Life Sciences). The FRET efficiency 39 was calculated as described previously 13.

### In situ proximity ligation assay (PLA)

Duolink In Situ Red Starter kit (MilliporeSigma) 40 was used to perform PLA according to the manufacturer's instructions. Briefly, murine T cells were incubated with primary antibody pairs (e.g., FoxO1 plus IKKβ, GLK plus PKCθ, or GLK plus IKKβ), followed by incubation with PLA probe-conjugated anti-mouse IgG and anti-rabbit IgG secondary antibodies. After ligation and amplification reactions, red-dot signals of the PLA probe pairs (depicting protein-protein interactions within 40 nm) were visualized by a confocal microscope (TCS SP5II, Leica Microsystems) 13.

### Immunofluorescence and confocal microscopy

Murine T Cells were fixed in cold methanol for 1 min. After permeabilization with fixation/permeabilization buffer (BD Bioscience) for 1 h, the cells were blocked with 5% bovine serum albumin for 1 h. The cells were incubated with rabbit anti-FoxO1 and mouse anti-IKKβ primary antibodies (1:200 dilution) for 16 h and then incubated with anti-rabbit IgG-CF488 and anti-mouse IgG-CF594 secondary antibodies (1:500 dilution), respectively, for 1 h. The secondary antibodies were purchased from Biotium. The fluorescence signals were analyzed using Leica TCS SP5II confocal microscope 13, 28.

### Single-cell separation, library construction and scRNA-seq

Murine T cells were purified from the spleen, inguinal lymph nodes, and mesenteric lymph nodes of wild-type and Lck-GLK Tg mice using negative selection. BD Rhapsody^TM^ Single-Cell Analysis System was used to generate the scRNA-seq library of T cells. SeqGeq version 1.6.0 (BD Biosciences) combined with R packaged Seurat plugin and ViolinBox plugin were used to analyze the cDNA quality, scRNA expression matrix, differentially expressed genes (DEGs), and gene ontology (GO) database.

### Statistical analyses

The statistical significance between two unpaired groups was analyzed using two-tailed Student's t test. *P* values of less than 0.05 were considered statistically significant. Symbols of *P* values represent *P < 0.05, **P < 0.01, ***P < 0.001, and n.s.: not significant.

## Supplementary Material

Supplementary figures and tables.
