# MAP4K3/GLK inhibits Treg differentiation by direct phosphorylating IKKβ and inducing IKKβ-mediated FoxO1 nuclear export and Foxp3 downregulation: Erratum

**DOI:** 10.7150/thno.125465

**Published:** 2026-01-30

**Authors:** Jyun-Ni Chi, Jhih-Yu Yang, Chia-Hsin Hsueh, Ching-Yi Tsai, Huai-Chia Chuang, Tse-Hua Tan

**Affiliations:** 1 Immunology Research Center, National Health Research Institutes, Zhunan, Taiwan.; 2 Department of Pathology & Immunology, Baylor College of Medicine, Houston, Texas, USA.

The authors regret that the original version of our paper, unfortunately, contained an unnecessary IKKβ blot in Figure 3C right panel. The purified IKKβ protein was purchased from SignalChem and was used for all Figure 3C experiments. The blot of IKKβ input control should be shown only once to avoid misunderstanding. The reorganized version of Figure 3C is shown below. In addition, individual blots of Figure 3A left panel were inadvertently flipped upside down. The correct version of Figure 3A is shown below.

The corrections made in this erratum do not affect the original data and conclusions. The authors apologize for any inconvenience that the errors may have caused.

## Figures and Tables

**Figure A:**
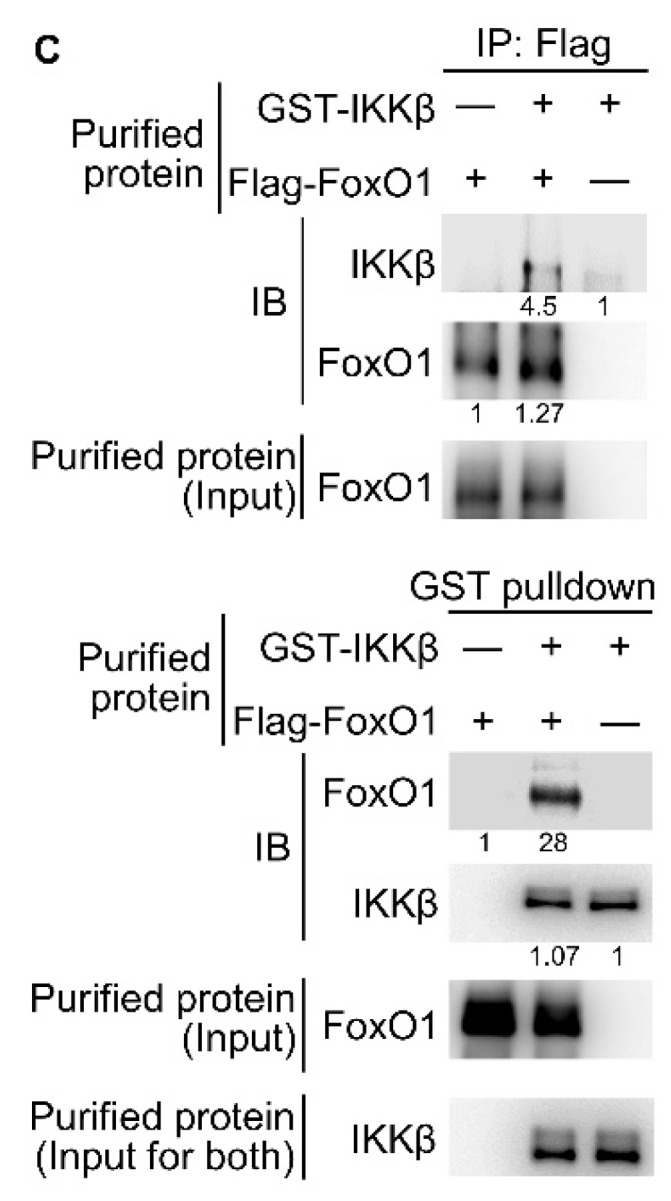
** Corrected Figure 3C** Purified Flag-tagged FoxO1 and GST-tagged IKKβ proteins were used for *in vitro* binding assay. The relative protein levels of the protein complexes determined by densitometry analysis were shown in the bottom of panels.

**Figure B:**
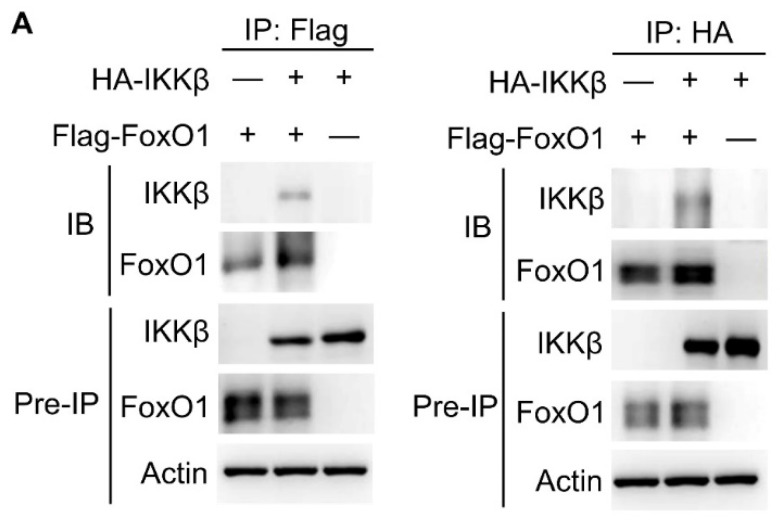
** Corrected Figure 3A** Co-immunoprecipitation experiments of Flag-tagged FoxO1 and HA-tagged IKKβ using lysates of HEK293T cells. IB, immunoblotting.

