# Supplementary material for: MAP4K3/GLK inhibits Treg differentiation by direct phosphorylating IKKβ and inducing IKKβ-mediated FoxO1 nuclear export and Foxp3 downregulation
Source: Theranostics. 2022 Jul 18;12(13):5744–60. doi: 10.7150/thno.72148 (PMC9373822; doi:10.7150/thno.72148)
Supplement: Supplementary file 1 — Supplementary figures and tables. [file thnov12p5744s1.pdf]

Supplementary information for

MAP4K3/GLK inhibits Treg differentiation by direct phosphorylating IKKβ and inducing IKKβ-mediated FoxO1 nuclear export and Foxp3 downregulation

Jyun-Ni Chi, Jhih-Yu Yang, Chia-Hsin Hsueh, Ching-Yi Tsai,
Huai-Chia Chuang, and Tse-Hua Tan

This PDF file includes:

Figure S1. Flow cytometry analysis of nTreg cells in vivo and iTreg cells in vitro.

Figure S2. IKKβ Ser733 phosphorylation is induced by GLK.

Figure S3. Phosphomimetic IKKβ (S733E) mutant does not induce FoxO3a phosphorylation at Ser644.

Figure S4. Confocal microscopy analysis of the interaction between the endogenous GLK and IKKβ proteins in T cells.

Figure S5. Foxp3 transcripts are not detectable in FoxO1-expressed $hGLK^+CD8^+$ T cells.

Figure S6. Foxp3 transcripts show no correlation with FoxO1 expression in $hGLK^-$ T cells.

Figure S7. Differentially expressed genes (DEGs) and nTreg-associated genes analysis of wild-type and Lck-GLK transgenic T cells.

Figure S8. Differentially expressed genes (DEGs) and nTreg-associated genes analysis of wild-type and Lck-GLK transgenic $FoxO1^+$ T cells.

Figure S9. nTreg-associated genes analysis of wild-type and Lck-GLK transgenic $CD8^+$ T cells with FoxO1 transcripts.

Figure S10. nTreg-associated genes analysis of wild-type and Lck-GLK transgenic $CD4^+$ T cells with FoxO1 transcripts.

Figure S11. Gene Ontology (GO) enriched pathway of differentially expressed genes (DEGs).

Table S1. List of differentially expressed genes (DEGs) in Lck-GLK transgenic T cells versus wild-type T cells.

Table S2. List of differentially expressed genes (DEGs) in FoxO1+ subpopulation of Lck-GLK transgenic T cells versus wild-type T cells.

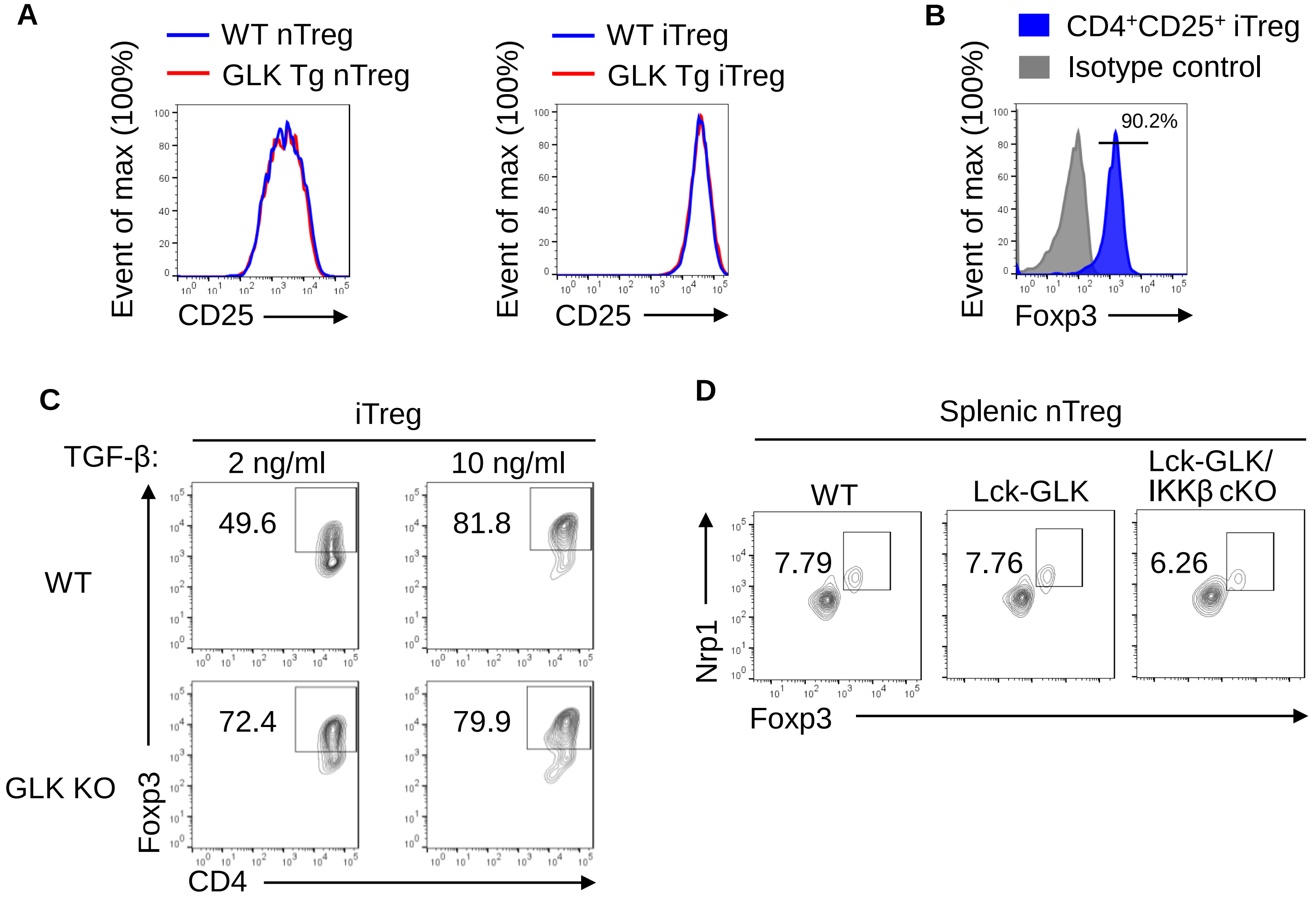

Figure S1. Flow cytometry analysis of nTreg cells in vivo and iTreg cells in vitro. (A) Histogram of CD25 expression gated on nTregs in vivo (left) or in vitro-differentiated $CD4^{+}Foxp3^{+}$ T cells (right). (B) Purity of $CD4^{+}Foxp3^{+}$ iTreg cells after positive selection using anti-CD25 antibody. (C) Flow cytometry analysis of $CD4^{+}Foxp3^{+}$ T cells in vitro-differentiated iTreg cells from wild-type (WT) and GLK-deficient mice using optimal concentration (10 ng/ml) and sub-optimal concentration (2 ng/ml) of TGF-β. (D) Representative contour plots of Foxp3 versus Nrp-1 in gated CD4+ T cells from the spleen of WT, Lck-GLK Tg, and Lck-GLK Tg/IKKβ cKO mice using flow cytometry analysis.

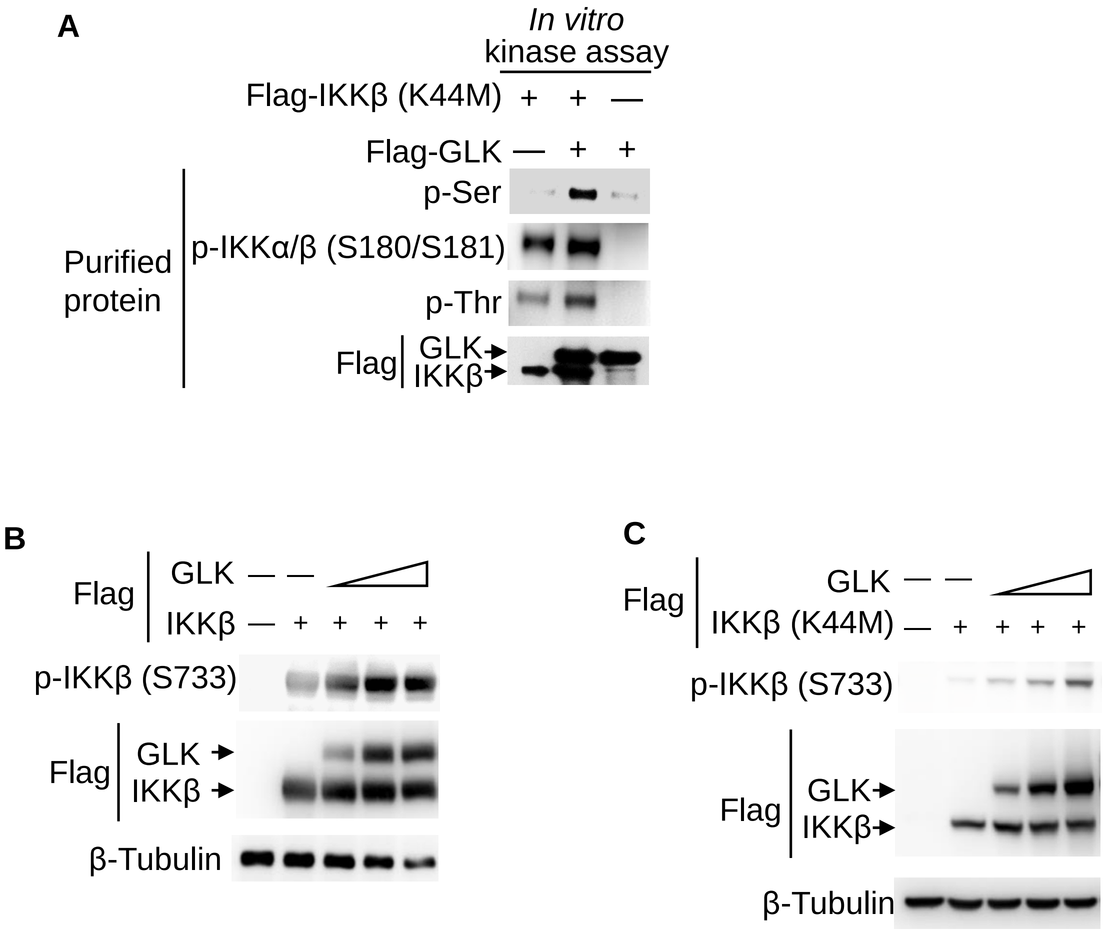

Figure S2. IKKβ Ser733 phosphorylation is induced by GLK. (A) *In vitro* kinase assays of immunopurified Flag-tagged GLK with Flag-tagged IKKβ kinase-dead (K44M) mutant proteins from individual HEK293T transfectants. (B) Immunoblotting analysis of phosphorylated (p-) IKKβ (Ser733), Flag-tagged GLK, Flag-tagged IKKβ, and β-Tubulin proteins in HEK293T cells. Cells were co-transfected with Flag-IKKβ alone or Flag-IKKβ plus increasing amounts of Flag-GLK plasmids. The higher basal phopho-Ser733-IKKβ levels of wild-type IKKβ (lane 2) may be due to cross-reactivity of this antibody to other autophosphorylation sites on wild-type IKKβ. To avoid the background signal of IKKβ autophosphorylation, we used Flag-IKKβ kinase-dead (K44M) mutant in the next panel. (C) Immunoblotting analysis of p-IKKβ (Ser733), IKKβ, GLK, and β-Tubulin proteins in HEK293T cells. Cells were co-transfected with Flag-IKKβ kinase-dead (K44M) mutant plus increasing amounts of Flag-GLK plasmids.

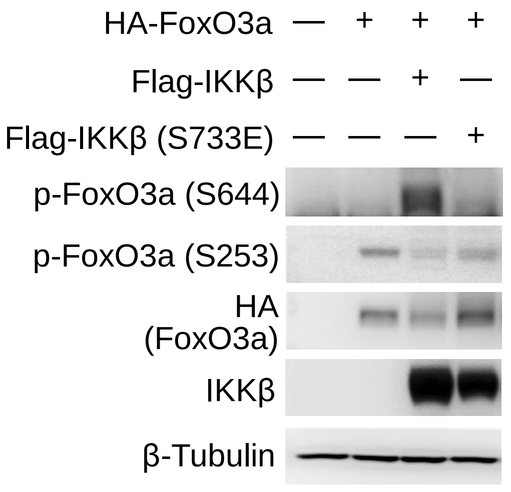

Figure S3. Phosphomimetic IKKβ (S733E) mutant does not induce FoxO3a phosphorylation at Ser644. Immunoblotting analysis of p-FoxO3a (S644), p-FoxO3a (S253), HA-tagged FoxO3a, and Flag-tagged IKKβ proteins in HEK293T cells co-transfected with HA-FoxO3a plus either Flag-IKKβ or phosphomimetic Flag-IKKβ (S733E) mutant plasmids.

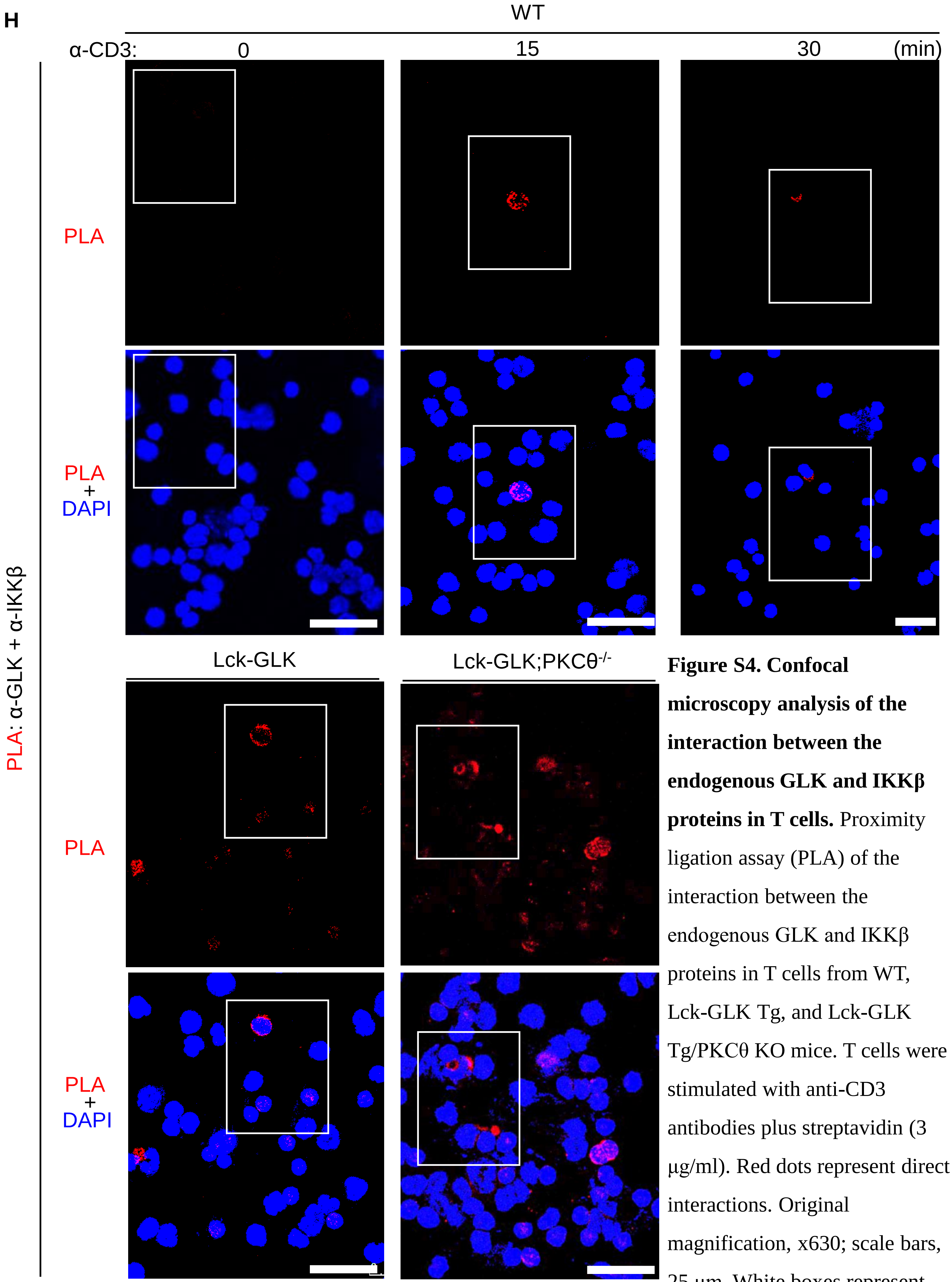

Figure S4. Confocal microscopy analysis of the interaction between the endogenous GLK and IKKβ proteins in T cells. Proximity ligation assay (PLA) of the interaction between the endogenous GLK and IKKβ proteins in T cells from WT, Lck-GLK Tg, and Lck-GLK Tg/PKCθ KO mice. T cells were stimulated with anti-CD3 antibodies plus streptavidin (3 μg/ml). Red dots represent direct interactions. Original magnification, x630; scale bars, 25 μm. White boxes represent the images shown in Figure 5H.

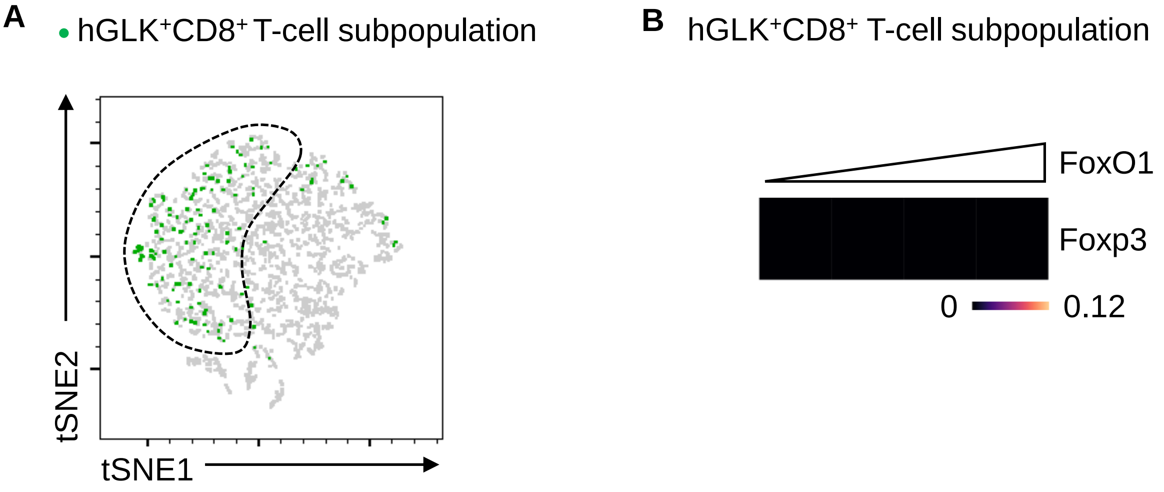

Figure S5. Foxp3 transcripts are not detectable in FoxO1-expressed hGLK$^+$CD8$^+$ T cells. (A) tSNE plot showing hGLK$^+$CD8$^+$ T-cell subpopulation (4.12%) in total T cells. **(B)** Heatmap showing the absence of Foxp3 expression in hGLK$^+$CD8$^+$ T-cell subpopulation with gradually elevated FoxO1 expression.

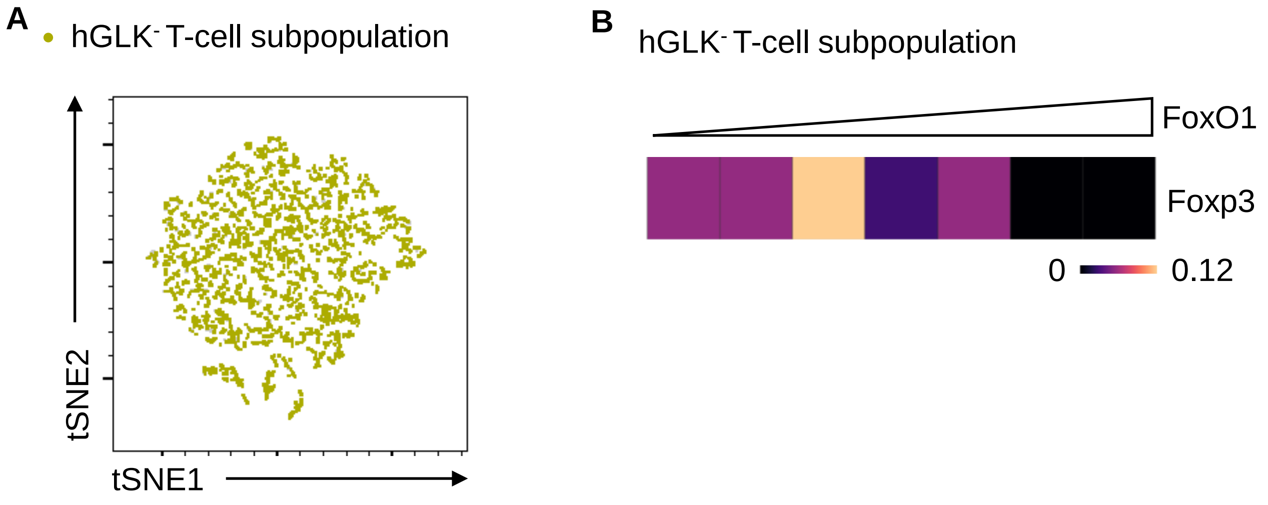

Figure S6. Foxp3 transcripts show no correlation with FoxO1 expression in hGLK$^-$ T cells. (A) tSNE plot showing hGLK$^-$ T-cell subpopulation (93.1%) in total T cells. **(B)** Heatmap showed that Foxp3 expression was gradually decreased in hGLK$^-$ T-cell subpopulation.

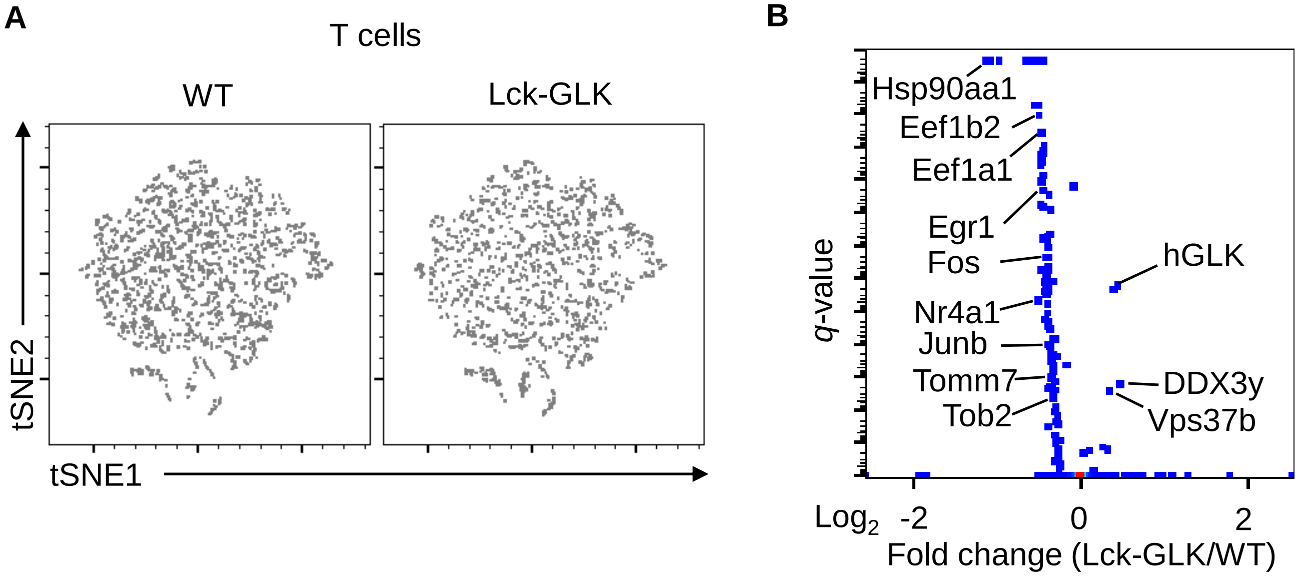

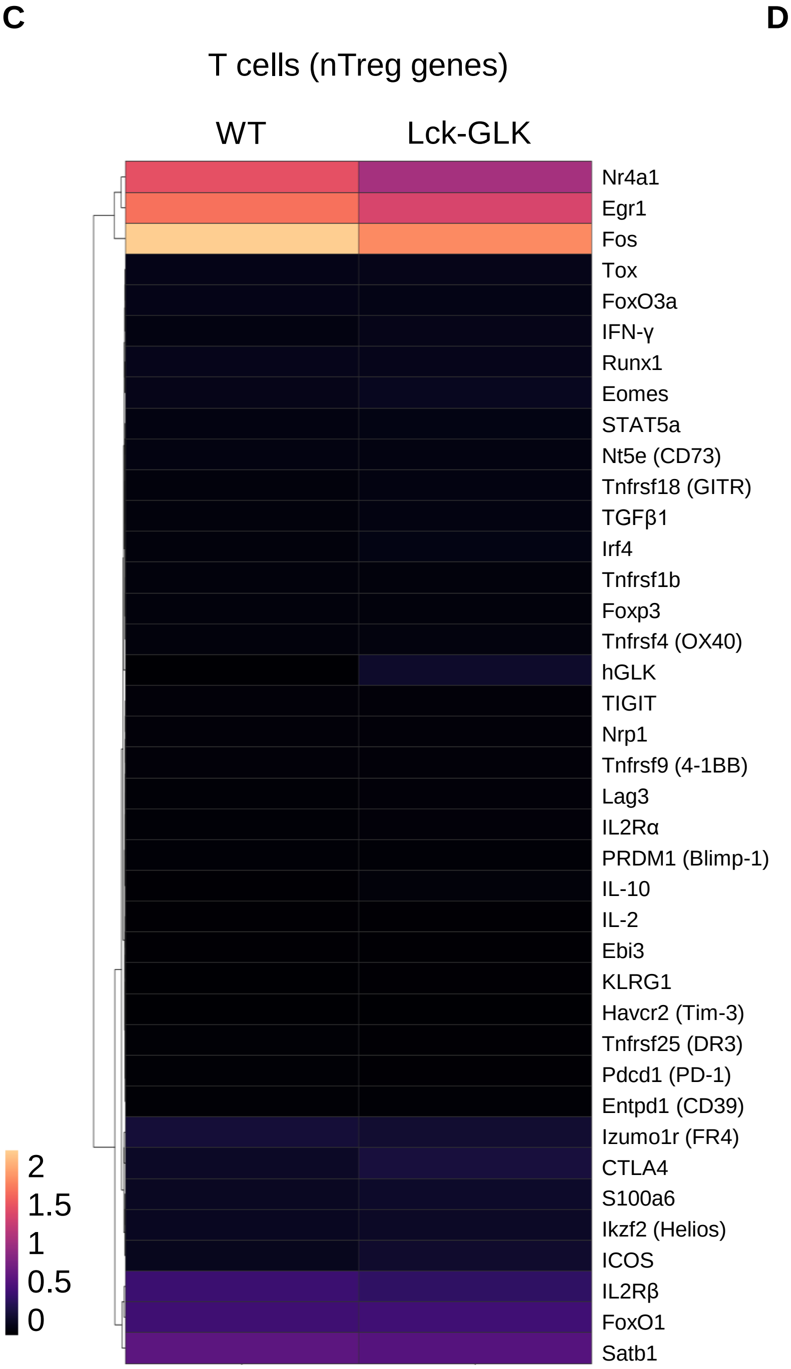

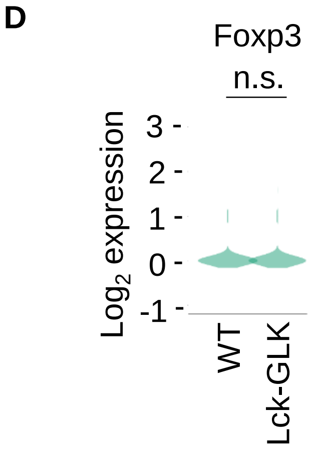

Figure S7. Differentially expressed genes (DEGs) and nTreg-associated genes analysis of wild-type and Lck-GLK transgenic T cells. (A) tSNE plots showing T-cell population in wild-type (WT) and Lck-GLK Tg mice. **(B)** Volcano plot showing the selected differentially expressed genes (DEGs) in Lck-GLK transgenic T cells versus WT T cells. **(C)** Heatmap showing the nTreg-associated transcripts expressed in T-cell population. **(D)** Violin plot showed that relative Foxp3 expression was unchanged in WT versus Lck-GLK transgenic T cells. n.s., not significant.

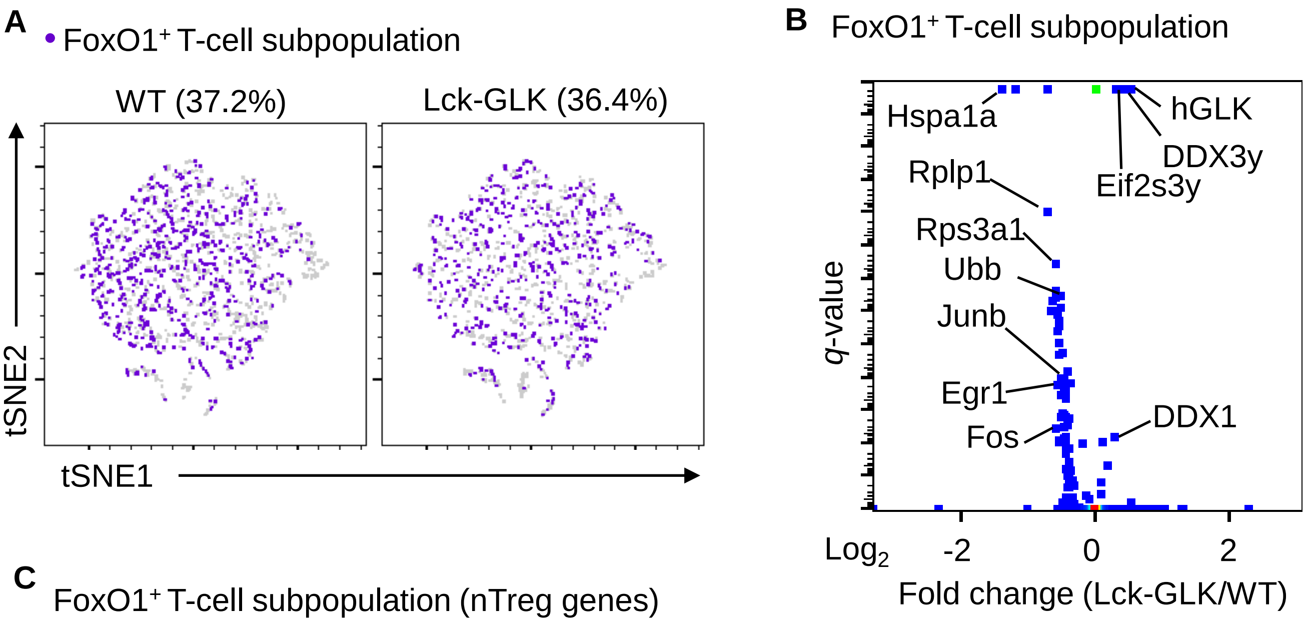

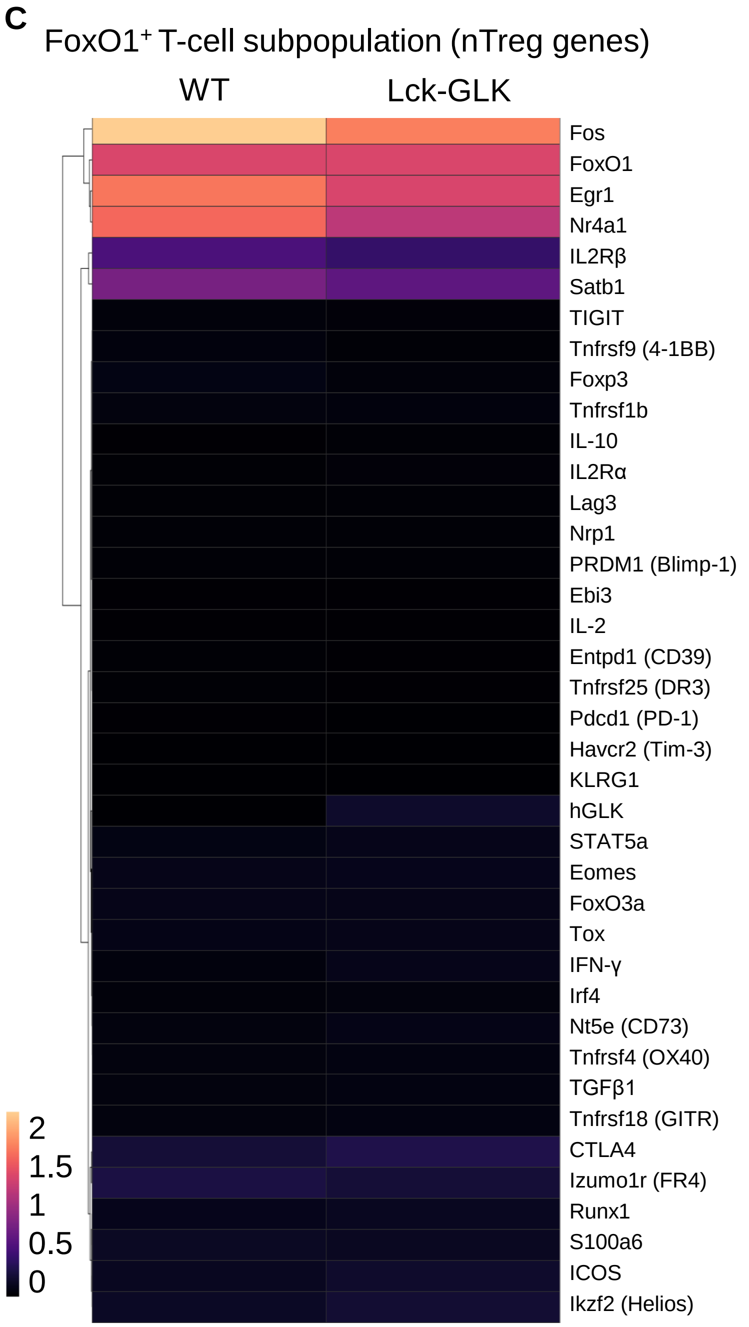

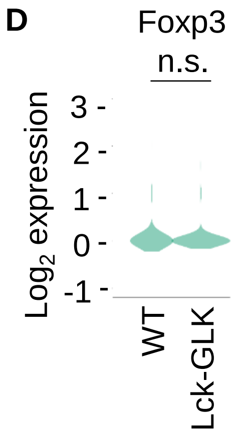

Figure S8. Differentially expressed genes (DEGs) and nTreg-associated genes analysis of wild-type and Lck-GLK transgenic FoxO1^{+} T cells. (A) tSNE plots showing FoxO1^{+} T-cell subpopulation in wild-type (WT) and Lck-GLK Tg mice. **(B)** Volcano plot showing the selected differentially expressed genes (DEGs) in FoxO1^{+} subpopulation of Lck-GLK transgenic T cells versus WT T cells. **(C)** Heatmap showing the Treg-associated transcripts in FoxO1^{+} T-cell subpopulation. **(D)** Violin plots showed that relative Foxp3 expression was unchanged in FoxO1^{+} subpopulation of WT versus Lck-GLK transgenic T cells. n.s., not significant.

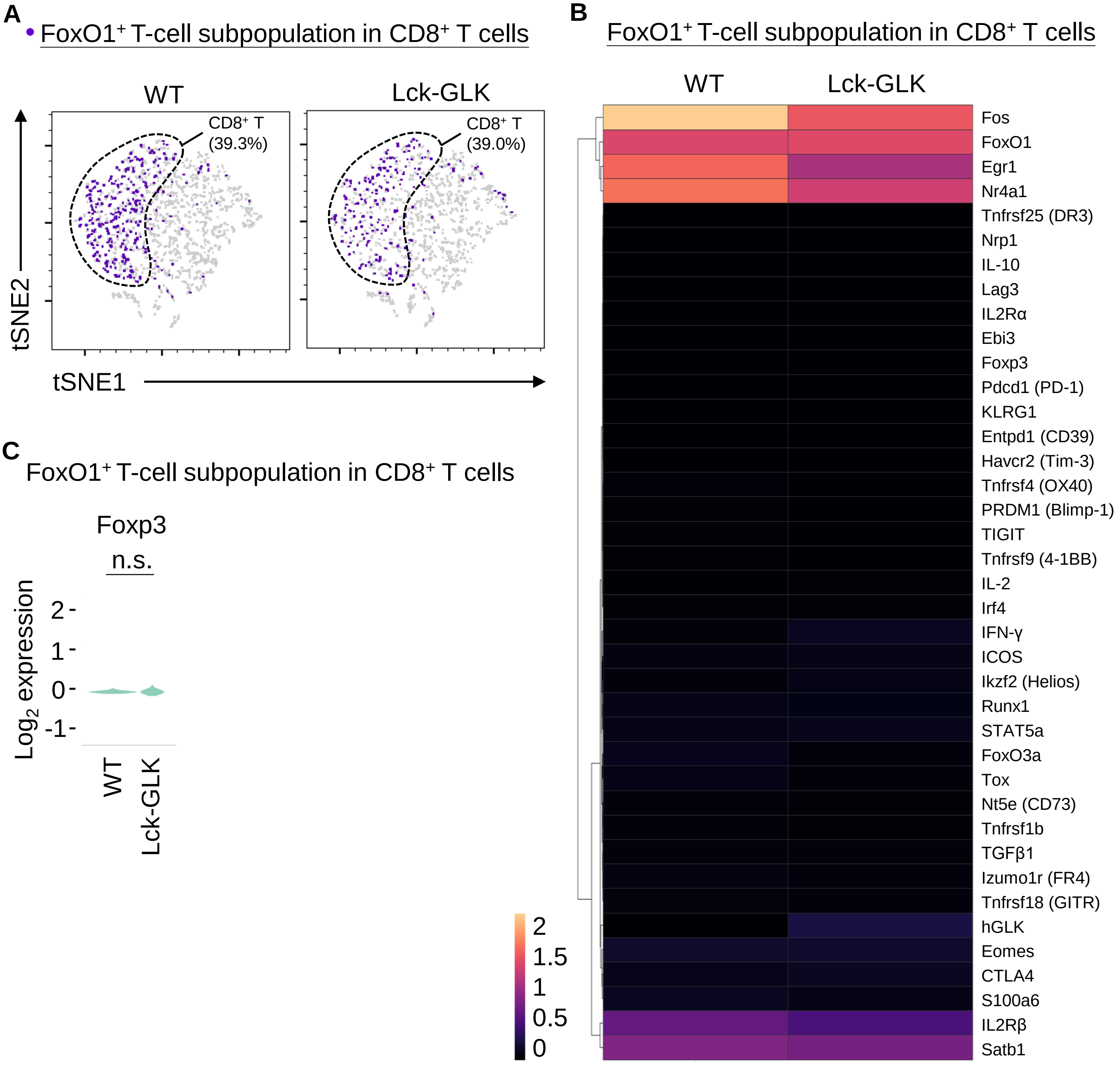

Figure S9. nTreg-associated genes analysis of wild-type and Lck-GLK transgenic CD8$^+$ T cells with FoxO1 transcripts. (A) tSNE plots showing FoxO1$^+$CD8$^+$ T-cell subpopulation in wild-type (WT) and Lck-GLK transgenic T cells. **(B)** Heatmap showing the Treg-associated transcripts expressed in FoxO1$^+$CD8$^+$ T-cell subpopulation. **(C)** Violin plots showed that relative Foxp3 expression was unchanged in FoxO1$^+$CD8$^+$ subpopulation of WT versus Lck-GLK transgenic T cells. n.s., not significant.

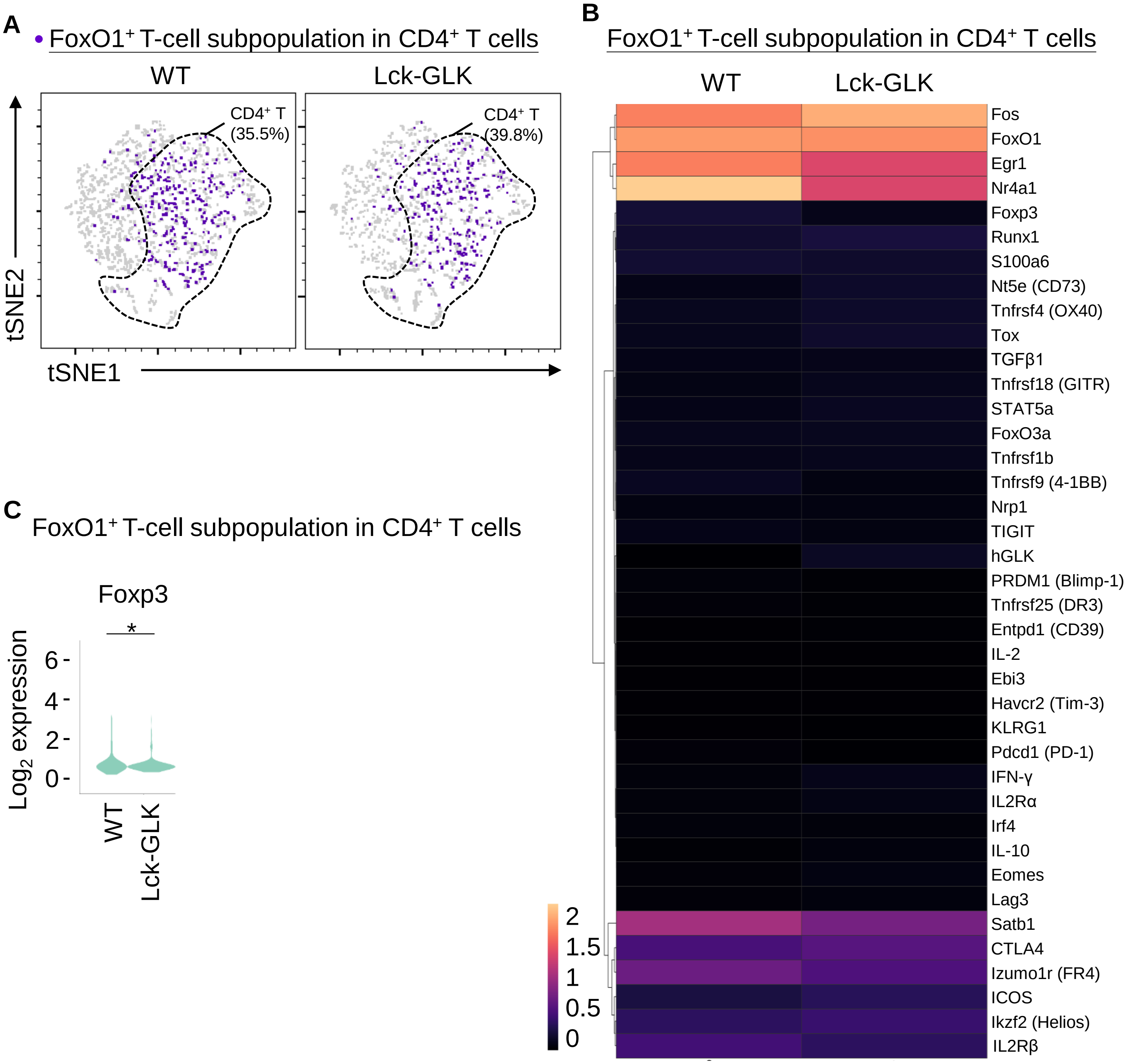

Figure S10. nTreg-associated genes analysis of wild-type and Lck-GLK transgenic CD4$^+$ T cells with FoxO1 transcripts. **(A)** tSNE plots showing FoxO1$^+$CD4$^+$ T-cell subpopulation in wild-type (WT) and Lck-GLK Tg mice. **(B)** Heatmap showing the Treg-associated transcripts expressed in FoxO1$^+$CD4$^+$ T-cell subpopulation. **(C)** Violin plots showed that Foxp3 expression levels were decreased in FoxO1$^+$CD4$^+$ subpopulation of Lck-GLK transgenic T cells compared to those of WT T cells. *$P < 0.05$ (two-tailed student's test).

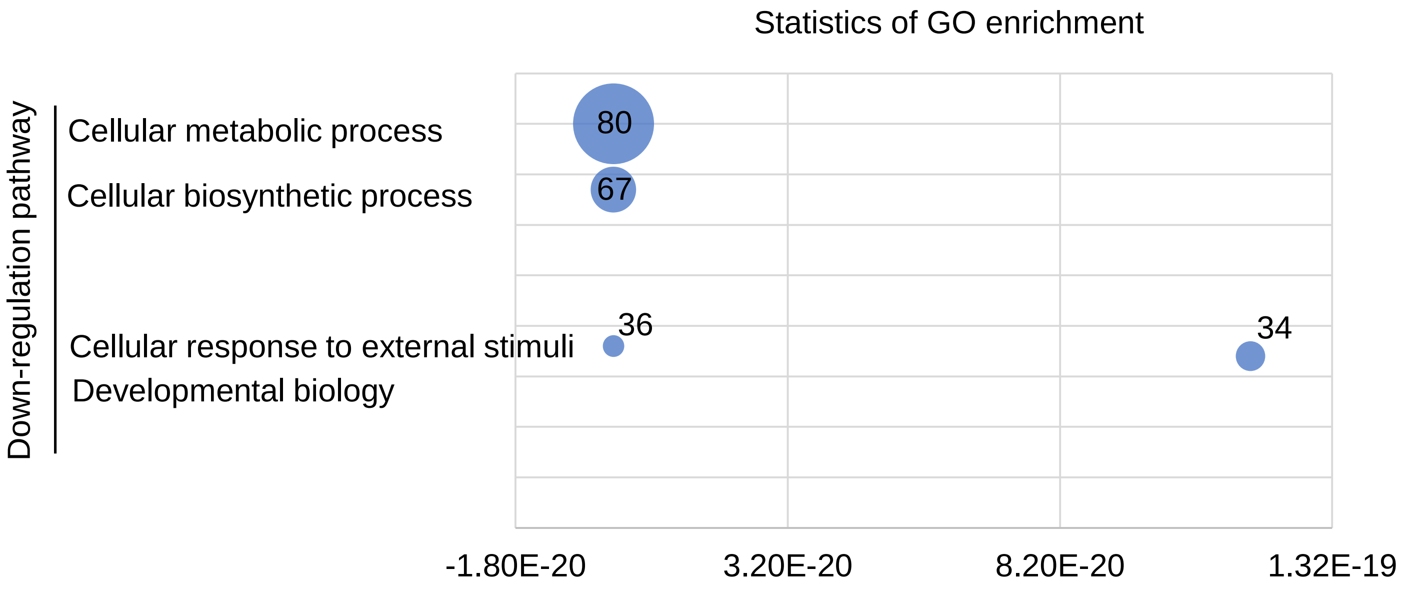

Figure S11. Gene Ontology (GO) enriched pathway of differentially expressed genes (DEGs). Bubble chart showing enrichment of down-regulated pathways in splenic T cells from Lck-GLK Tg mice. Pathways belonging to different classifications are listed on the left of the plot. Varied numbers of genes enriched in individual pathways are presented by different diameter sizes and numbers for individual dots. Numbers represent amounts of DEGs in each pathway.

Gene	Fold change	*q*-value
Calr	0.91	4.00E-04
CD3	0.88	4.40E-05
CD52	0.88	6.00E-04
DUSP2	0.87	8.00E-04
Eef1a1	0.86	3.12E-11
Eef1b2	0.82	3.12E-12
Eef1g	0.89	7.00E-04
Egr1	0.88	2.00E-09
Fos	0.88	4.00E-06
Fosb	0.94	5.90E-05
Gm11808	0.87	1.30E-05
Gm2000	0.9	1.00E-02
Hsp90aa1	0.8	1.76E-13
Hsp90ab1	0.8	1.76E-13
Hspa1a	0.67	1.76E-13
Hspa1b	0.68	1.76E-13
Hspa8	0.84	1.76E-13
Junb	0.88	3.60E-06
Ms4a6b	0.89	3.00E-03
Npm1	0.88	4.00E-04
Nr4a1	0.83	2.00E-07
Pabpc1	0.88	9.00E-05
Ppp1r15a	0.82	1.30E-13
Ptprc	0.88	1.50E-05
Rack1	0.88	1.00E-04
Rpl10	0.89	7.90E-06
Rpl11	0.9	9.00E-03
Rpl12	0.85	4.20E-07
Rpl13	0.86	8.40E-07
Rpl17	0.84	1.70E-13
Rpl18	0.87	3.30E-07
Rpl18a	0.85	1.50E-09
Rpl21	0.89	1.00E-03
Rpl22	0.86	9.70E-07
Rpl23	0.84	6.20E-11
Rpl23a	0.87	5.40E-06
Rpl26	0.87	9.30E-06
Rpl27a	0.88	1.50E-05
Rpl28	0.88	2.80E-05
Rpl3	0.88	8.50E-05
Rpl30	0.89	1.00E-03
Rpl31	0.89	2.00E-04
Rpl32	0.88	1.00E-03
Rpl34	0.86	8.00E-08
Rpl35	0.9	2.00E-03
Rpl35a	0.85	3.40E-10
Rpl36a	0.86	1.00E-05
Rpl37	0.85	1.10E-10
Rpl37a	0.084	1.50E-11
Rpl38	0.85	2.10E-09
Rpl39	0.82	1.70E-13
Rpl4	0.85	2.50E-08
Rpl4l	0.91	3.00E-03
Rpl5	0.84	4.30E-11
Rpl6	0.85	2.00E-10
Rpl8	0.9	1.00E-02
Rpl9	0.83	1.70E-13
Rpl9-ps6	0.86	1.40E-07
Rplp0	0.84	4.60E-11
Rplp1	0.79	1.70E-13
Rplp1	0.79	1.70E-13
Rplp2	0.89	1.00E-03
Rps11	0.87	6.40E-06
Rps12	0.83	3.90E-12
Rps13	0.87	2.30E-08
Rps14	0.86	1.80E-10
Rps15	0.85	7.30E-09
Rps15a	0.85	9.30E-09
Rps16	0.84	1.70E-13
Rps17	0.86	3.30E-09
Rps18	0.85	3.20E-08
Rps19	0.87	2.90E-06
Rps2	0.9	9.00E-03
Rps20	0.86	3.70E-07
Rps21	0.89	7.00E-04
Rps23	0.86	2.80E-08
Rps24	0.84	1.50E-10
Rps26	0.86	7.40E-09
Rps27	0.87	2.90E-07
Rps27a	0.89	2.40E-05
Rps27rt	0.88	3.50E-06
Rps28	0.84	1.90E-11
Rps29	0.88	7.90E-07
Rps3	0.87	5.90E-07
Rps3a1	0.84	1.70E-13
Rps4x	0.86	1.30E-08
Rps5	0.84	1.50E-11
Rps6	0.86	2.90E-06
Rps7	0.82	1.70E-13
Rps8	0.88	4.00E-04
Rps9	0.87	8.10E-07
Rpsa	0.85	7.90E-09
Thy1	0.84	3.90E-07
Tmsb4x	0.87	2.30E-08
Tob2	0.86	7.00E-03
Tomm7	0.9	2.00E-03
Tpt1	0.87	2.50E-10
Trbc2	0.88	4.00E-04
Ubb	0.84	1.70E-13
Ubc	0.86	1.5E-11

Table S1. List of differentially expressed genes (DEGs) in Lck-GLK transgenic T cells versus wild-type T cells.

Gene	Fold change	*q*-value
CD3	0.85	3.00E-04
CD52	0.8	9.00E-03
Eef1a1	0.83	3.20E-07
Eef1b2	0.79	9.50E-07
Egr1	0.88	1.00E-04
Fos	0.87	1.00E-03
Hsp90aa1	0.79	4.30E-07
Hsp90ab1	0.78	1.76E-13
Hspa1a	0.61	1.76E-13
Hspa1b	0.66	1.76E-13
Junb	0.83	9.70E-05
Ppp1r15a	0.81	3.30E-03
Rpl10	0.87	3.60E-02
Rpl12	0.83	3.00E-04
Rpl17	0.84	1.00E-04
Rpl18	0.86	8.40E-03
Rpl18a	0.83	1.90E-05
Rpl22	0.83	8.00E-03
Rpl23	0.83	1.00E-04
Rpl26	0.85	7.00E-03
Rpl27a	0.85	1.00E-02
Rpl28	0.86	8.00E-03
Rpl3	0.85	2.00E-03
Rpl34	0.85	1.00E-03
Rpl35a	0.83	1.80E-06
Rpl37	0.81	1.10E-06
Rpl37a	0.82	2.00E-06
Rpl38	0.86	7.00E-03
Rpl39	0.82	3.90E-06
Rpl4	0.82	1.00E-04
Rpl5	0.81	3.30E-07
Rpl6	0.87	1.40E-02
Rpl9	0.81	8.70E-07
Rpl9-ps6	0.83	1.00E-04
Rplp0	0.85	1.00E-03
Rplp1	0.78	8.99E-10
Rps11	0.84	7.00E-03
Rps12	0.82	2.60E-06
Rps13	0.84	1.60E-05
Rps14	0.85	2.00E-04
Rps15	0.83	8.40E-06
Rps15a	0.83	1.00E-04
Rps16	0.85	1.00E-04
Rps20	0.85	6.00E-03
Rps24	0.82	1.00E-06
Rps26	0.84	1.00E-03
Rps27a	0.86	2.00E-03
Rps28	0.84	1.00E-04
Rps3a1	0.81	3.20E-08
Rps4x	0.87	3.00E-02
Rps5	0.84	1.00E-03
Rps6	0.86	7.00E-03
Rps7	0.81	2.10E-07
Rps8	0.85	1.00E-02
Rps9	0.85	1.00E-03
Rpsa	0.84	1.00E-03
Thy1	0.82	7.00E-03
Tpt1	0.87	6.30E-05
Ubb	0.83	6.70E-07
Ubc	0.86	2.00E-04

Table S2. List of differentially expressed genes (DEGs) in FoxO1^{+} subpopulation of Lck-GLK transgenic T cells versus wild-type T cells.
